# Robust Inversion of Time-Resolved Data via Forward-Optimization in a Trajectory Basis

**DOI:** 10.1021/acs.jctc.2c01113

**Published:** 2023-05-02

**Authors:** Kyle Acheson, Adam Kirrander

**Affiliations:** † EaStCHEM, School of Chemistry and Centre for Science at Extreme Conditions, University of Edinburgh, David Brewster Road, Edinburgh EH9 3FJ, United Kingdom; ‡ Physical and Theoretical Chemistry Laboratory, Department of Chemistry, University of Oxford, South Parks Road, Oxford OX1 3QZ, United Kingdom

## Abstract

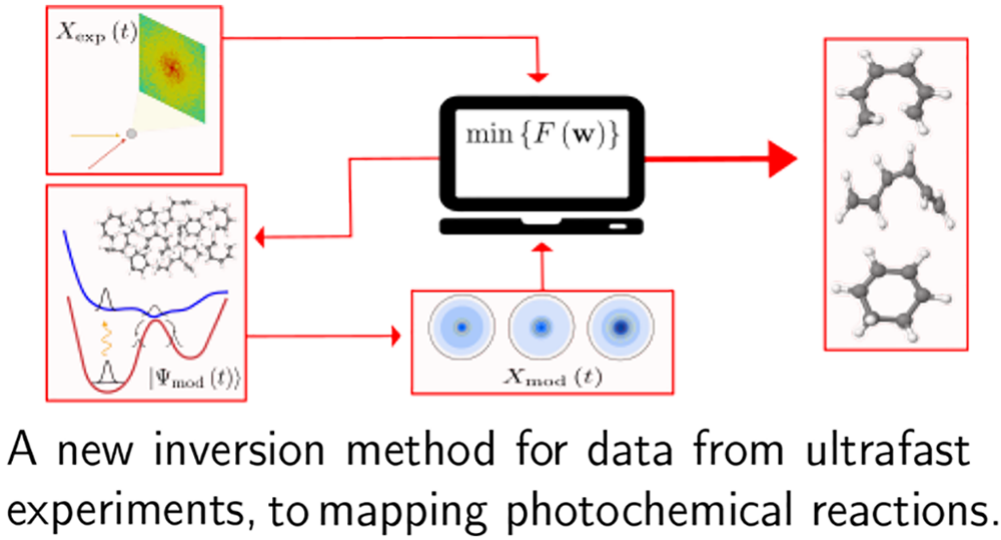

An inversion method for time-resolved data from ultrafast experiments is introduced, based on forward-optimization in a trajectory basis. The method is applied to experimental data from X-ray scattering of the photochemical ring-opening reaction of 1,3-cyclohexadiene and electron diffraction of the photodissociation of CS_2_. In each case, inversion yields a model that reproduces the experimental data, identifies the main dynamic motifs, and agrees with independent experimental observations. Notably, the method explicitly accounts for continuity constraints and is robust even for noisy data.

## 1 Introduction

Inverse problems are important across science and engineering,^1−3^ and have a long history at the borderline between physics and mathematics.^4^ They concern the determination of key target characteristics from an observed set of outputs, for instance determining the shape of a vibrating membrane from its spectrum, or reconstructing a three-dimensional object from a photograph. Important applications appear in engineering, medical imaging, and material characterization.^1−3,5,6^

The inverse problem is fundamentally different from the, arguably more common, forward problem, in which an observable or a property is predicted by direct numerical solution of physically motivated equations. In particular, the inverse problem is generally mathematically ill-posed and underdetermined, suffering instability and a lack of unique solutions. The overall aim is thus to find a robust method capable of identifying a physically reasonable model commensurate with the data. Suitable techniques include regularization, objective functionals, and a broad cross-section of computational methods including machine learning, neural networks, genetic algorithms, stochastic and statistical methods, and optimization schemes.

For molecular structure determination the inverse problem is central. In general, the structure of a molecule is underdetermined by the experimental data and the molecular model is optimized subject to auxiliary constraints, which play a key role. Examples include the fitting of spectroscopic data to a Morse oscillator or Dunham coefficients in a diatomic,^7^ and more broadly, the use of molecular geometry constraints when refining a model of molecular structure in X-ray crystallography or NMR,^8^ as well as supplementary experimental and computational data in gas-phase electron diffraction structure determination.^9^ Importantly, while the constraints on e.g. bond distances and angles are well-known for ground-state equilibrium structures, for excited-state dynamics such constraints are *much* harder to define. The approach presented in this paper provides a solution to this conundrum.

In recent years, ultrafast imaging of photoexcited molecules have developed rapidly, with experiments increasingly capable of tracking molecular dynamics on fundamental time scales, observing phenomena such as vibrations, bond breaking, or charge transfer.^10−12^ The techniques include spectroscopy (e.g., TRPES),^13−16^ ultrafast electron diffraction (UED),^17,18^ ultrafast X-ray scattering (UXS),^19,20^ Coulomb explosion imaging (CEI),^21^ and others. Extracting a detailed time-dependent molecular model from this data is extremely challenging. In terms of structural dynamics, a minimal model would consist of a sequence of structures, while a complete model would provide the full temporal evolution of the molecular wave function with all associated time-dependent nuclear and electronic distributions and state populations.

The challenges involved in inversion of ultrafast data are so significant that in most cases inversion is never attempted. Instead, interpretation of the experiment relies on side-by-side comparison between theory and experiment. Ideally, this is done in terms of a comparison between predicted observables and experimental data. However, such comparison only provides a qualitative understanding and leaves little recourse if agreement between experiment and theory is not achieved. Inversion algorithms capable of providing a rapid, first-order, assessment of the observed dynamics would carry great value for experimental progress, providing a degree of decoupling between theory and experiment and insight into, for instance, what range of molecular models might be commensurate with the observations.

Thus, efforts to tackle the inverse problem for ultrafast dynamics are intensifying. One recently developed approach for the interpretation of ultrafast X-ray scattering data samples a large pool of randomly generated molecular structures in order to identify the “best fit” structure,^12,22,23^ while genetic algorithms have been proposed to invert electron diffraction data by exploring molecular structure via consecutive *in silico* mutations.^24−28^

There are also approaches that try to improve structure determination by scattering via the inclusion of phase retrieval algorithms, which requires at least partial alignment of the molecules.^29−34^ All of these methods are applicable to both static and time-dependent scenarios, but inversion methods that specifically target dynamic situations have also been conceived. This includes machine learning approaches that use a variational recurrent neural network trained on temporally correlated frames^35^ and an approach that systematically perturbs the molecular structure at each step, starting with the well-known initial structure at time zero.^36^

In this paper, we present a detailed discussion of a new method for the inversion of time-dependent data which explicitly accounts for the time-evolution. This ensures continuity, accounts for the correct initial (and final) structures, and provides as realistic as possible constraints for excited state dynamics. The method proceeds by optimizing the weights of semiclassical trajectories from quantum molecular dynamics simulations against experimental data. In essence, this matches the simulations to the experimental observations. The approach is general, and provides a platform for merging data from several complementary experiments, with initial focus on the analysis of experimental data from ultrafast X-ray and electron scattering experiments.^37−39^ The aim of the paper is to provide the first unified and general presentation of this methodology, and to critically evaluate its performance, establish best-practice, and explore avenues for improving the methodology further.

## 2 Theory

### 2.1 Forward Optimization

The time evolution of any observable *X*(*t*) can be calculated from the molecular wave function |Ψ(*t*)⟩ via a forward mapping *M*,
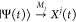
1where the index *j* identifies the type of measurement, which could be anything from photoelectron spectra to electron diffraction or Coulomb explosion imaging. The observable *X*^*j*^(*t*) may be resolved with respect to several implicit variables, for instance photoelectron kinetic energy and angular distribution. In contrast to the inverse problem, the forward mapping is mathematically well-conditioned and does not suffer stability or underdetermination issues. We therefore proceed to tackle the inverse as a forward optimization problem,^37−39^ where our goal is to find a model molecular wave function  that yields predicted observables  that reproduce the experimental observables . The calculation of the observable consists of two steps,
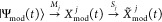
2where the first step is the forward mapping *M*_*j*_ from eq 1, which produces the theoretically predicted signal, and a second *apparatus mapping* step *S*_*j*_ which replicates the effect of the measurement apparatus on the data, for instance due to limited time resolution. Such distortions are unavoidable despite that the experimental data  will have been through extensive preprocessing to remove known artifacts and distortions. This approach allows the iterative refinement of the model wave function in light of the constraints placed on the observable by a given experimental setup. Crucially, the wave function is refined globally over all time and not just with respect to the sampling of the initial conditions of the trajectories.

The optimization proceeds by modifying the model wave function until close agreement between the experimental and predicted data is achieved, as measured by the target function *F*,
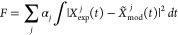
3where the index *j* runs over the different types of experiments included. In practice it is common for the integration over time *t* to be replaced by a summation over a temporal grid {*t*_*i*_}. The α_*j*_ is a regularisation factor that must be included when data from several different types of experiments is considered. The factor is determined from the numerical profile of each of the different data sets, and scales data so that it can be combined in a balanced manner.

The reminder of the paper is organized such that Section 2.2 presents the reference molecular wave function, Section 2.3 presents the parametrized model wave function and the target function that result from the model function, and Section 2.4 discusses the forward mapping with an emphasis on scattering experiments. In Section 3, the range of applicable numerical optimization techniques are discussed, as well as the *S*_*j*_ mapping to match the predicted observables to the experiment. The preprocessing of the experimental data is touched upon in Section 4 on data treatment and in the Supporting Information (SI). In the Results, Section 5, two applications to recent ultrafast electron diffraction and ultrafast X-ray scattering data are examined in detail and the convergence and the resulting interpretation are assessed.

### 2.2 Molecular Wave Function

The time evolution of the molecular wave function |Ψ(*t*)⟩ is governed by the time-dependent Schrödinger equation,
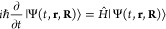
4where *Ĥ* is the molecular Hamiltonian and **r** and **R** the electronic and nuclear coordinates. The molecular wave function can be expanded in the Born–Huang form as,^40^
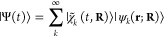
5where  are time-dependent nuclear wavepackets which propagate on electronic eigenstates  that depend parametrically on the nuclear coordinates **R**. (In the following, **r** and **R** will be dropped from the equations.) In practice, the expansion in eq 5 is truncated to include only the *N*_*s*_ electronic states visited during the dynamics of interest.

A wide range of numerical techniques to solve eq 4 exist. Accurate methods such as numerical grid propagators^41,42^ and multiconfigurational time-dependent Hartree (MCTDH)^43^ require precalculated potential energy surfaces which are not feasible for most molecules of interest. An alternative is direct dynamics (dd) methods that expand the molecular wave function by classical or semiclassical trajectories. Examples include surface hopping (SH), *ab initio* multiple spawning (AIMS), *ab initio* multiconfigurational Ehrenfest (AIMCE), and direct dynamics variational multiconfigurational Gaussians (dd-vMCG).^44−47^ In a general form, the direct dynamics wave function  can be expressed as,
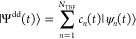
6with *c*_*n*_(*t*) the expansion coefficient for each of the *N*_TBF_ trajectory basis functions (TBFs) given by,
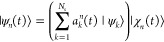
7where the parentheses contains the electronic states |ψ_*k*_⟩ and their populations . The nuclear basis functions |χ_*n*_(*t*)⟩ follow phase-space trajectories (**R**_*n*_(*t*), **P**_*n*_(*t*)) where **R**_*n*_(*t*) and **P**_*n*_(*t*) are the nuclear positions and momenta, respectively. The equations of motion, which govern the trajectories, populations, and auxiliary coefficients such as for instance wavepacket width coefficients, are different for each method. Thus, the details of each specific dd wave function will vary. For instance, the nuclear wavepacket is a Gaussian^48^ in all methods except SH where it is a δ-function, for SH and AIMS only one electronic state is occupied by each TBF at any given time, while Ehrenfest methods such as AIMCE occupy several states simultaneously, in AIMS (AIMCE) spawning (cloning^49^) increases the number of TBFs as the simulation progresses, *etc*. Overall, it is important that the simulations used to generate the basis for the model wave function in Section 2.3 below are as accurate as possible, both in terms of the electronic structure and propagation method, and that their accuracy is assessed carefully with respect to known data from e.g. spectroscopic measurements.

### 2.3 Model Wave Function

We adapt the wave function in eq 6 as our parametrized model wave function by rescaling the expansion of the TBFs. In effect, the TBFs can be thought of physically reasonable constraints on a system far from equilibrium, which in addition automatically fulfill continuity requirements. The resulting model function is given by,
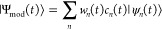
8where *w*_*n*_(*t*) are the weights for the TBFs which are adjusted to bias the theoretical model toward the experiment, subject to a normalization condition .

If observables are taken to only depend on the nuclear coordinates and are calculated in the diagonal zeroth-order bracket-averaged Taylor expansion (BAT) approximation (see ref (50)), as we will do here, the coefficients *w*_*n*_(*t*) and *c*_*n*_(*t*) can be taken to be time-independent with the normalization straightforwardly given by *∑*_*n*_*w*_*n*_ = 1 for *c*_*n*_ ≡ 1. For SH wave functions, this is always the case. The target function in eq 3 then becomes a function of the time-independent weights .

As a final aside, we note that the current approach can also be used when the target is best described by a density matrix, by augmenting the model with additional model wave functions weighted by their population factors.

### 2.4 Forward Mapping

The forward optimization exploits that the direct problem, i.e. the forward mapping,
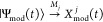
9is mathematically well-defined and has stable solutions. For each type of experimental observable the mapping *M*_*j*_ will be different and based on different theoretical approximations and computational techniques. For instance, there is an extensive body of work on the prediction of time-resolved photoelectron spectra^51,52^ with available techniques ranging from approximate Dyson orbitals calculations^53,54^ to the highly accurate R-matrix method.^55^ The method used to calculate the X-ray scattering and electron diffraction signals in the current paper will be discussed in Section 3. Generally, it is important that the forward mapping is sufficiently accurate to allow meaningful comparison to the experimental data. Approximate methods for observable calculation must be justified on a case-by-case basis for a given experimental resolution. If highly accurate calculations are not computationally viable, care must be taken and features not captured by the approximation isolated prior to optimization.

## 3 Computational Methods

### 3.1 Trajectory Basis Functions

#### 3.1.1 1,3-Cyclohexadiene Ring-Opening

**Figure 1:**
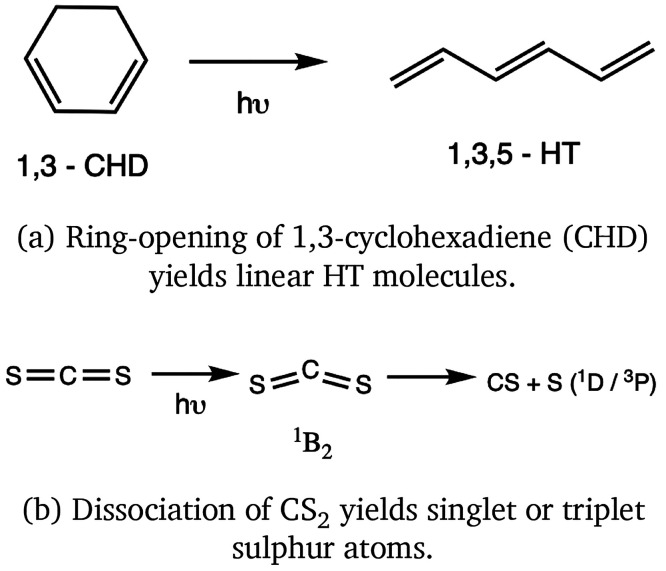
Schematic of the two photochemical processes probed in the experiments for which data is inverted in this paper, via a basis of trajectory basis functions (TBFs).

The ring-opening reaction of 1,3-cyclohexadiene (CHD) to 1,3,5-hexatriene (HT), shown schematically in Figure 1a, is a prototypical Woodward–Hoffmann photoinduced electrocyclic reaction.^56^ It has been the target for a large number of pioneering time-resolved experiments that include UXS, UED, and time-resolved spectroscopies.^37,38,53,56−65^ Upon absorption of a 267 nm photon the molecule undergoes a π → π* transition to the steeply sloped 1B electronic state, glancing the conical intersection with the 2A electronic state while staying on the adiabatic potential energy surface. Passage through the conical intersection to the electronic ground state returns the molecule to the original ring-closed CHD or breaks a C–C bond to yield the ring-open HT. A distribution of various *cis* (*Z*)/*trans* (*E*) HT isomers are observed given the high internal energy of the system.

Semiclassical trajectories, TBFs, for the dynamics are calculated using the *ab initio* Ehrenfest (AIMCE) method.^37,38^ The electronic structure calculations use the *ab initio* package MOLPRO,^66^ which supplies the forces and the nonadiabatic couplings at 3SA-CAS(6,4)-SCF/cc-pVDZ level of theory for the ground and excited states. A set of 100 TBFs, propagated for 200 fs, with initial conditions sampled from a Wigner distribution in the Franck–Condon region of equilibrium ground state CHD provide the basis for the trajectory-fitting procedure. It is important that the sampling is generous and allows for a broad range of trajectories to be considered in the optimization.

#### 3.1.2 CS_2_ Photodissociation

The photodissociation of CS_2_, shown schematically in Figure 1b, has been the subject of numerous time-resolved experiments.^11,39,67−77^ Upon excitation to the ^1^*B*_2_ state, rapid bending and stretching vibrational motion is observed. More complex excited state dynamics ensues, exhibiting a striking competition between internal conversion (nonadiabatic couplings) and intersystem crossing (spin–orbit coupling), resulting in two dissociation channels that yield either S(^1^*D*) or S(^3^*P*) sulfur atoms, with the triplet product dominant.

Semiclassical trajectories (TBFs) to be used in the forward optimization are calculated using the SHARC surface-hopping package.^78,79^ The trajectories start on the optically bright ^1^Σ_*u*_^+^ state with a total energy corresponding excitation by a 200 nm pulse and initial coordinates sampled from the ground state Wigner distribution. The forces and nonadiabatic couplings are calculated using SA8-CASSCF(10,8)/SVP electronic structure theory using MOLPRO^66^ and 197 trajectories are propagated for 1 ps. Further information can be found in SI Section 3.2.

### 3.2 Scattering Observables

The forward mapping required to compare the model to the UXS and UED data is summarized below. Although scattering signals can be calculated from first-principles for gas-phase molecules^80−88^ and recent X-ray scattering measurements have demonstrated that given sufficient accuracy different electronic states can be resolved,^10,89−91^ the experimental data considered in this paper can be modeled using the significantly simpler independent atom model (IAM), which approximates the scattering as a summation over the coherent scattering from isolated atoms.

The instantaneous energy-integrated total scattering cross section into the solid angle *d*Ω is then,^50,92,93^
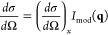
10where **q** = **k**_0_ – **k**_1_ is the scattering vector expressed in terms of the wave vectors of the incoming and outgoing photon or electron. (The scattering (momentum transfer) vector **q** is denoted **s** in electron scattering.) For X-ray scattering, the prefactor (*dσ*/*d*Ω)_*x*_ is the differential Thomson scattering cross section (*dσ*/*d*Ω)_Th_, here made to include the  polarization factor, while for electron scattering it is the Rutherford cross section (*dσ*/*d*Ω)_Rh_ which here includes the scaling factor *s*^–4^.^94,95^ Note that the expression above does not account for the duration of the X-ray pulse, which is accounted for via the temporal convolution discussed in Section 3.3.

Using the diagonal BAT approximation and assuming time-independent expansion coefficients norm **w** = 1, the scattering intensity *I*(**q**) can be calculated as,
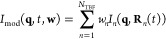
11More general expressions that account for the full wave function in eq 6, including the nonlocal nature of the individual TBF nuclear wavepackets, have been derived previously.^50^ Continuing with the current simplified form, sufficient for our present needs, yields the scattering intensity as,
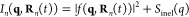
12where *S*_inel_(*q*) is the inelastic scattering, which is independent of molecular geometry and given by an incoherent summation over the atomic contributions,
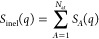
13with *N*_at_ the number of atoms and *S_A_*(*q*) the inelastic atomic form factors. The corresponding elastic contribution is given by the form factor *f*(**q**, **R**_*n*_(*t*)),
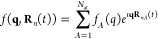
14where *f*_*A*_(*q*) are the atomic form factors and **R**_*nA*_(*t*) the position vector for atom *A* in trajectory *n*. Both *f*_*A*_(*q*) and *S*_*A*_(*q*) are tabulated.^96^ The form factors for electron scattering are , where *Z*_*A*_ is the atomic number and *f*_*A*_ the atomic form factor for X-ray scattering.^94,95^ For high energy electron scattering, notably MeV-UED, it is sometimes necessary to use form factors with relativistic corrections.^97,98^

When the target is a gas of anisotropic molecules, rotational averaging of eq 14 results in,^99^
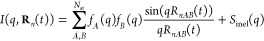
15with the distance *R*_*nAB*_(*t*) = |**R**_*nA*_(*t*) – **R**_*nB*_(*t*)| between atoms *A* and *B* in trajectory *n*.

### 3.3 Apparatus Mapping

In this section we discuss the *apparatus mapping S*_*j*_ in eq 2 that is required to match the forward mapped signal *X*_*mod*_^*j*^(*t*) from eq 9 to the experimentally observed signal. The apparatus mapping provides a mechanism for accounting for systematic issues with the experimental data, as long as these are identified.

#### 3.3.1 Temporal Alignment and Convolution

In the scattering experiments, the time-zero is roughly calibrated by the instrument, with the exact time-zero inferred from the observed data. The absence of independent validation means that one must check the alignment of the temporal axes in the experiment and the model. (Absolute changes on the experimental time-axis are accurate, trivially so for the model/theory.) We define the relationship between the experimental and model time axes as *t*′ = *t* + *t*_0_, where *t*′ is the experiment, *t* the model, and *t*_0_ the time shift. The temporal alignment *t*_0_ is one of the global parameters optimized.

A Gaussian convolution in the temporal domain is included as,
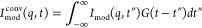
16where *G*(*t*) = *b*_*c*_ exp(−*a*_*c*_*t*^2^) mimics the instrument response function, with the normalization constant given as  and τ_*c*_ the full-width half-maximum (fwhm). The convolution mainly equates to the cross-correlation of the pump and the probe, effectively compensating for the δ(*t*)-pulse excitation approximation used to generate the TBFs. In practice, the instrument response function also accounts for other limits on the temporal resolution, such as temporal jitter.

Finally, we note that the experimental resolution with respect to the momentum transfer *q* is such that no convolution of the model is required. The amount of structural information in the signal is limited by the *q*-range, *q* ∈ [*q*_min_, *q*_max_], measured in the experiment.^38,93^

#### 3.3.2 Percent Difference Signal

The experimental signal is considered in the percent difference form to minimize systematic multiplicative errors,
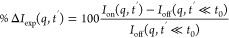
17where *I*_on_(*q*,*t*) is the optically pumped “*laser-on*” signal and  is the static *’laser-off’* reference signal measured at delay times *t*′ ≪ *t*_0_. The theoretical equivalent is calculated from the model wave function  and defined as,
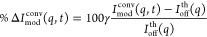
18where the excitation fraction γ scales the intensity according to the implicit degree of excitation. The theoretical “*laser-off*” signal *I*_off_^th^(*q*) is calculated from a suitable reference geometry, or more accurately using a Wigner distribution of the system in its ground state at the equilibrium geometry. In some cases, it may be necessary to modify the definition in eq 18 to scale the signal by the ratio of the integrated intensity of the “*laser-on*” and “*laser-off*” signal, or replace the uniform excitation fraction γ with a *q* dependent excitation fraction γ(*q*). Such modifications are discussed in the SI.

### 3.4 Target Function and Confidence Matrix

The experimental percentage difference signal %Δ*I*_*exp*_(*q*,*t*) and the signal predicted from the model %Δ*I*_*mod*_(*q*,*t*,*w*) give the target function introduced in eq 3 the following specific form,
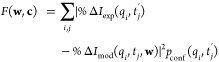
19where  are the normalized trajectory weights ∥**w**∥ = 1, and **c** consists of additional global parameters to be optimized, such as e.g. the excitation fraction γ and the time-shift *t*_0_. In principle, more complicated global parameters designed to offset shortcomings in the quality of the TBFs could be included, such as time-warping to offset inaccuracies in the kinetic energy. The double sum runs over all experimental data points, identified by *q*_*i*_ momentum transfer and the temporal coordinates *t*_*j*_^′^ = *t*_*j*_ + *t*_0_.

To avoid overfitting of inherently noisy experimental data, the target function includes a confidence matrix. The matrix  weights data points, identified by their value of the momentum transfer *q*_*i*_ and time bin *t*_*j*_^′^, according to the experimental confidence in the accuracy of that data point. For instance, data points subject to poor statistics or systematic errors are given smaller weight while points that are known or expected to be accurate are weighted accordingly. The exact form of  depends on the data set, as discussed in the SI.

If needed, one can define a confidence threshold *p*_conf_^min^, such that all points  are zero, thus excluding them from the optimization. The advantage of excluding poor quality points from the optimization must be balanced against the reduced size of the experimental data set.

### 3.5 Global Optimization

In the ideal scenario, the experimental signal has sufficient quality (high temporal resolution, large *q*-range, and excellent signal-to-noise) that simultaneous optimization of all model parameters is possible. Since the number of global parameters is small, the most straightforward approach is to optimize the trajectory weights **w** for fixed global parameters **c**, and to determine from a scan of reasonable values of **c** the best overall solution.

For each particular **c**, a pool of *N*_init_ initial weights {**w**}_init_ are Monte Carlo sampled with the values of the individual weights in each initial set *w*_*i*_ ∈ [0, 1]. The target function given in eq 19 is then minimized utilizing a nonlinear trust-region reflective least-squares algorithm for each initial set. Among the optimizations that converge to a local minimum, within the tolerance constraints, the best minimum is considered a candidate for the global minimum. The number of initial conditions *N*_init_ generated in the weight space is increased until apparent convergence is achieved. The convergence of the optimization with respect to *N*_init_ is discussed in SI, Section 4.1. In challenging cases, an iterative approach using more targeted sampling can be used, which is discussed in the SI.

### 3.6 Two-Step Optimization

When the experimental data is of lower quality, more stable solutions are found via a two-step optimization procedure. The first step ensures that global parameters, such as *t*_0_ and τ_*c*_, are determined as accurately as possible before, in the second step, optimization of the trajectory weights is attempted.

#### 3.6.1 Step 1: Global Parameters

First, we identify the strongest features in the data by inspection of the confidence matrix . This allows us to minimize the negative effects of noise on the optimization. Selecting the strongest feature, we define the net integrated percentage difference signal as,
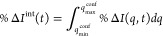
20where *q*_min_^conf^ and *q*_max_^conf^ are the bounds on the section of highest confidence. The global parameters **c** are then optimized against this integrated signal, %Δ*I*_exp_^int^(*t*). Since this step must be performed independently from the optimization of the trajectory weights **w**, we assume that the *t* ≈ *t*_0_ wave function can be approximated by an equally weighted sum of TBFs. This is reasonable given the limited dispersion and dephasing in the wavepacket at early times.

The *t*_0_ is inherently linked to τ_*c*_ and γ since the pulse width τ_*c*_ affects the onset of the signal and γ scales the strength of the signal. Therefore, for different combinations of *t*_0_ and τ_*c*_, the sum of square error between the experimental and model integrated difference signal in eq 20 is minimized with the γ excitation fraction as the free parameter.

#### 3.6.2 Step 2: TBF Weights

The second step in the optimization takes the best values of  from the previous step with the goal of identifying the optimal trajectory weights **w**. Since the γ in step one is determined on the assumption of equally weighted TBFs, the parameter γ is reoptimized alongside the weights **w** in this second step. If the final value of γ diverges significantly from its initial value, this may indicate that the parameters determined in the first step are not optimal. In order to ensure self-consistency, it is advisible to repeat the second step optimization for several different sets of global parameter values  which correspond to good fits in the first step. The procedure is complete once the best sets of these values from step one and two agree, and when the target function *F*(**w**, **c**) has converged.

### 3.7 Metrics of Fit Quality

In addition to the target function *F*(**w**, **c**), it is useful to have other measures of the quality of the fit. The improvement in overall agreement between the unweighted and weighted model wave function can be quantified by the relative absolute error (RAE),
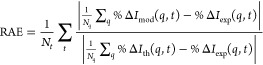
21where *N*_*t*_ is the number of time steps, *N*_*q*_ the number of points in *q*, and %Δ*I*_th_(*q*, *t*) the theoretical signal calculated from the theoretical wave function not subjected to any bias or optimization. Thus, quantifying how much better the optimized model fits the experimental observable in comparison to the unoptimized, values below 1 reflect an improvement. The RAE measure, as defined above, is independent of the size of the *N*_*t*_ × *N*_*q*_ grid, therefore allowing comparisons between different fits. Another helpful metric is the root mean squared error (RMSE) defined as,
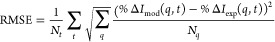
22

Particular solutions can also be characterized using the variance of the *N*_TBF_ weights from a single optimization, i.e. the spread of weights resulting from one of the *N*_init_ initial conditions (typically the initial condition that yields the best solution for a specific set of global parameters), defined as,
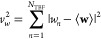
23where ⟨**w**⟩ is the mean of the weights. In addition, to compare different solutions we define the distance *D*_*b*_ from the overall best set of optimized weights,
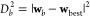
24which describes the distance for a particular solution *b* from the best set of optimized weights.

## 4 Experimental Data

### 4.1 Ultrafast X-ray Scattering

**Figure 2:**
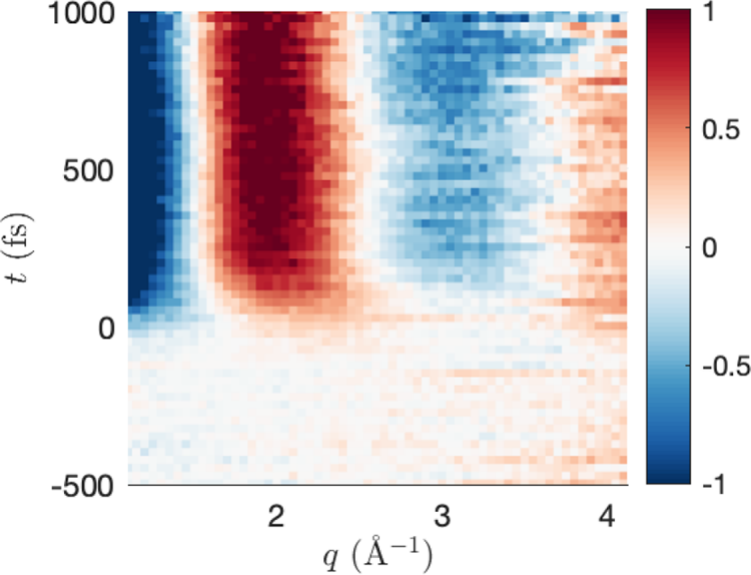
A subsection of the experimental UXS signal %Δ*I*_exp_(*q*, *t*) for CHD from ref (38). The experimental signal has been shifted in time so that it is centered at *t* = 0, instead of the *t*′ = −110 fs along the original raw experimental time axis.

The ultrafast X-ray scattering (UXS) data for the ring-opening reaction of CHD shown in Figure 2 is taken from ref (38) (further details in the SI, Section 3.1). The confidence matrix *p*_conf_ in eq 3 in this case is based on the number of photon hits per frame (SI, eq S7). Due to a long interaction region in these experiments, we include a *q*-dependent excitation fraction γ(*q*) which is indirectly optimized by allowing it to be uniformly scaled by a factor *x* such that it yields the scaled excitation fraction γ_*x*_(*q*) (for more details see the SI). Accounting for the convolution of the signal as in eq 16 and the subsequent temporal binning into bins of size Δ*t* = 25 fs, the total length of the signal used becomes 275 fs.

The quality of the experimental data allows a one-step global optimization in which we scan over a range of *t*_0_ values and *x* scaling factors, where *t*_0_∈[−38,–14] and *x*∈[0.7,1.3]. The duration of the pump and probe pulse were measured from experiment as 60 and 30 fs respectively, which fixes the value of τ_*c*_.

### 4.2 Ultrafast Electron Diffraction

The ultrafast electron diffraction (UED) data for the photodissociation of CS_2_ is taken from ref (39) (further details in the SI, Section 3.2). The data does not support the real-space pair distribution function (PDF) analysis obtained by a sine-transform of the modified scattering signal Δ*sM* that is common in UED (see SI, Section 1). Instead, the signal is evaluated in the percent difference form as per eq 17.

**Figure 3:**
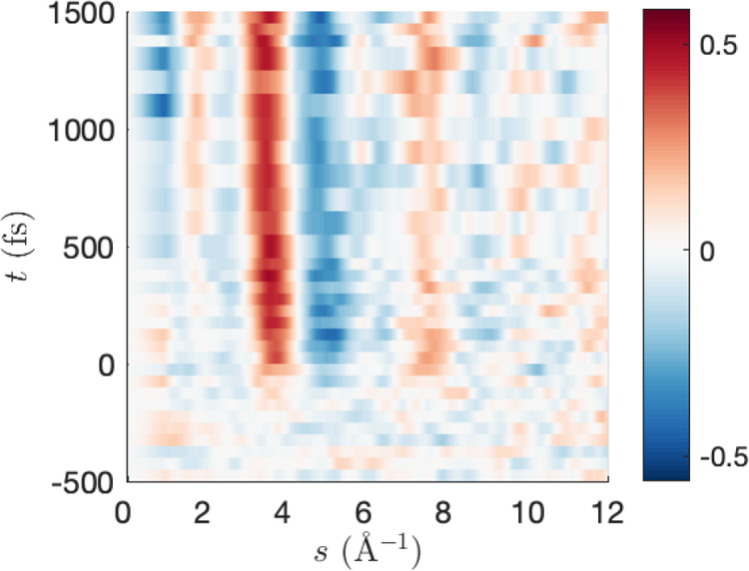
A subsection of the UED signal %Δ*I*_exp_(*s*, *t*) for CS_2_ from ref (39). The signal is initially centered on *t*′ = −120 fs.

The experimental signal %Δ*I*_exp_(*s*, *t*) in Figure 3 displays a strong enhancement band (red) next to a strong depletion band (blue) in the range 3.5 < *s* < 6.0 Å^–1^. Also note less intense enhancement/depletion features for *s* < 2 Å^–1^ which appear at later times and which correlate with the onset of strong dissociation.

Given the noise and limited temporal resolution in the data, we carry out a two-step optimization. In the first step, we fit *t*_0_ using eq 20 with bounds [*s*_min_, *s*_max_] = [2.8, 4.2] Å^–1^. This procedure is repeated in the range *t*_0_∈[−16,83] fs and τ_*c*_∈[150,250] fs. The best global parameters are then used in the determination of the weights **w**. Initial conditions for **w** are generated both using unbiased Monte Carlo sampling and using the biased iterative sampling procedure described in SI, Section 2. In either case, the confidence matrix *p*_conf_ is based on estimated experimental standard deviations (see SI, eq S9).

## 5 Results and Discussion

### 5.1 CHD Ring-Opening (UXS)

**Figure 4:**
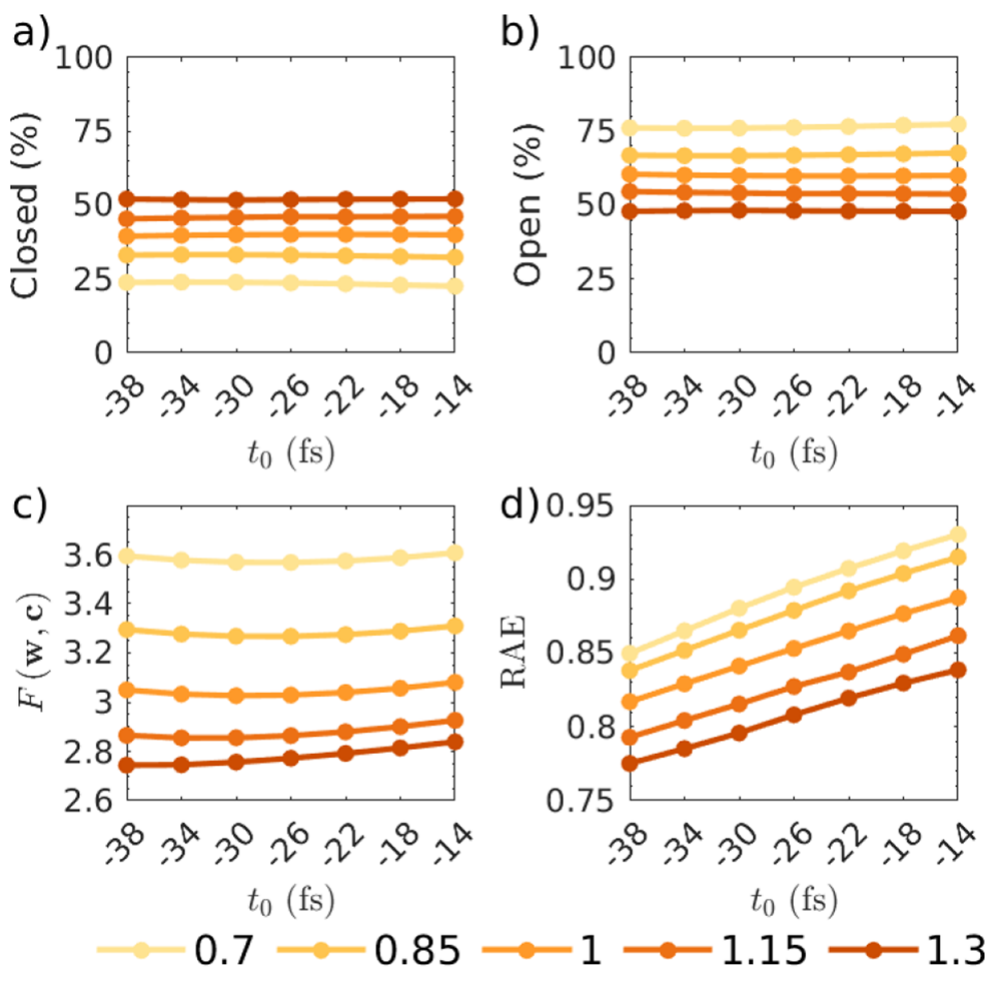
Convergence in the CHD optimization, showing the a) ring-closed fraction, b) ring-open fraction, c) the target function and d) the relative absolute error (RAE) as a function of *t*_0_. Each color represents a different value of the scaling factor *x*, ranging from 0.7 to 1.3.

For the CHD reaction, the data is of sufficient quality that a global one-step optimization is feasible. Optimization yields the best fit parameters , with a RAE of 0.775. Recall, the scaling factor *x* is optimized as to uniformly scale the *q* dependent excitation fraction such that, γ_*x*_(*q*) = γ(*q*)*x*. The values of γ(*q*) and the resulting optimized γ_*x*_(*q*) over the available *q* range, can be seen in SI Figure S19. The convergence with respect to *x* and the *t*_0_ shift in Figure 4 shows that more negative *t*_0_ shifts and larger *x* result in lower values of the target function *F*(**w**,**c**) and better RAE’s (with the trend more pronounced for higher *x*). Generally, *F*(**w**,**c**) is more sensitive to *x* than *t*_0_.

The change in the fraction of ring-open trajectories is small as *t*_0_ changes, which is unsurprising given that the majority of the ring-opening occurs in a concerted fashion within the first 140 fs.^56^ Current literature values are found in the range 40–60%.^56^ We note that higher values of *x* redistribute some of the ring-open weights to ring-closed weights. Since one does not physically expect the fraction of open to closed trajectories to change as a function of the excitation fraction, this is an artifact which stems from the fact that the percent difference signal is stronger for ring-open molecules than ring-closed, due to the reference equilibrium CHD structure being ring-closed. In principle, this could be addressed by a different choice of reference structure.

**Figure 5:**
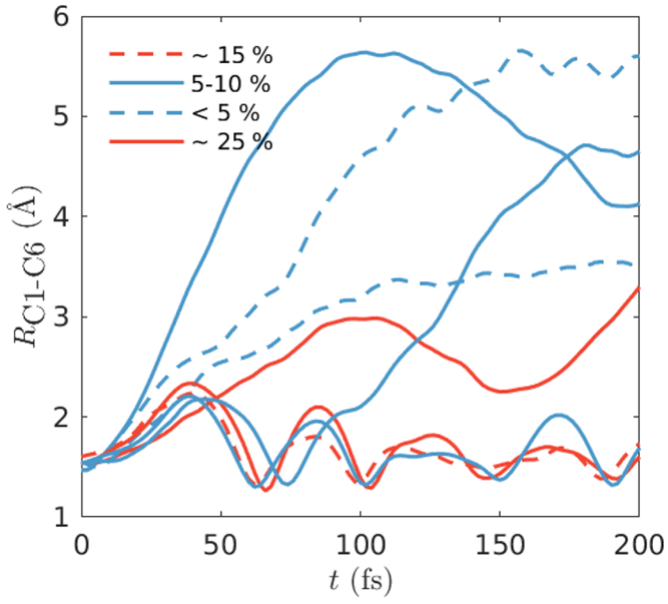
C_1_–C_6_ distances for the eight CHD trajectories with a weight greater than 1%. There are three distinct classes of trajectories: ring-closed, direct ring-opening and indirect (delayed) ring-opening.

The eight dominant trajectories in the final solution are summarized in Table 1. These account for almost all the weights. The trajectories can be visualized in terms of their characteristic C_1_–C_6_ bond distance as shown in Figure 5. Three main families of trajectories are observed: direct ring-opening, a slower indirect ring-opening which undergoes several C_1_–C_6_ stretches before breaking the bond, and finally ring-closed paths with initially strong oscillations in the C_1_–C_6_ bond which are damped out as the energy disperses across all motions. In total, the ring-opening and closed trajectories have a weight of 52% and 48%, respectively.

**Table 1:** Weights of the Dominant Trajectories from the Forward Optimization for CHD, along with Their Type

Weight (%)	Type
28.4	open (indirect)
27.3	closed
15.1	closed
9.70	closed
8.70	open
6.05	open (indirect)
3.38	open
1.35	open

The ground state HT has several *cis-/trans-* (*Z*)/(*E*) isomers. Out of the 48% HT product, we predominately observe the presence of cEc-HT (8.7%) and cZc-HT (34.5%) isomers. Due to the length of the 275 fs temporal window used in the fit it is no surprise we can not clearly detect the tZt-HT isomer. However, we note that we observe a small fraction (3.4%) with a configuration between cZt and tZt. With data available over a longer temporal range, one could better refine the ground state dynamics and eventually observe as the system settles into a thermal equilibrium between different HT isomers.

**Figure 6:**
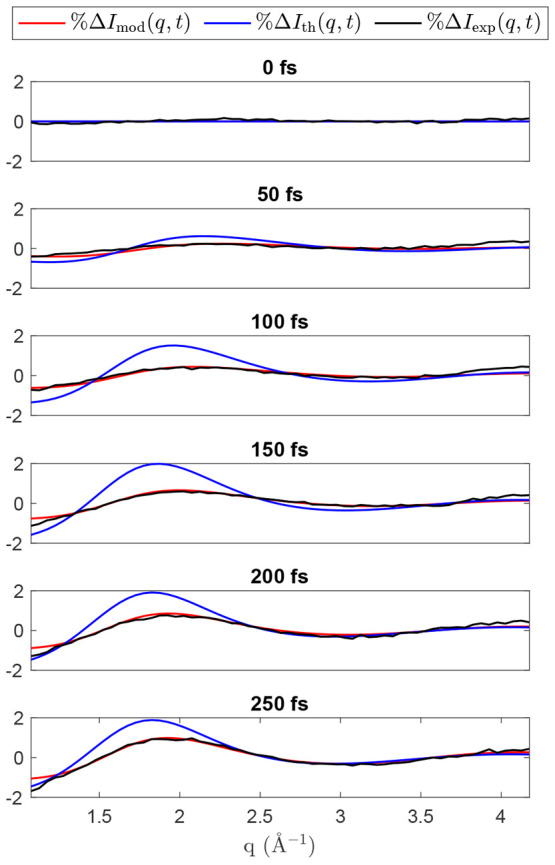
Comparison of the experimental %Δ*I*_exp_(*q*, *t*), unoptimized %Δ*I*_th_(*q*, *t*), and optimized %Δ*I*_mod_(*q*, *t*) signals. The corresponding heatmaps over all time delays can be seen in SI Figure S17.

Figure 6 shows the result of the optimization on the percentage difference signal. It is clear the optimized model shows better agreement with experiment both qualitatively in terms of the signal, and quantitatively in terms the metrics previously discussed. The signal corresponding to the optimized model shows improved agreement in terms of intensity and the center of the main peak around 2 Å^–1^. In the unoptimized case, this feature appears to shift to lower *q* values by a marginally larger amount as time evolves. Furthermore, note the improved agreement of the optimized model to the experiment in the *q* range of 2.75–3.25 Å^–1^ between 100 and 150 fs.

**Figure 7:**
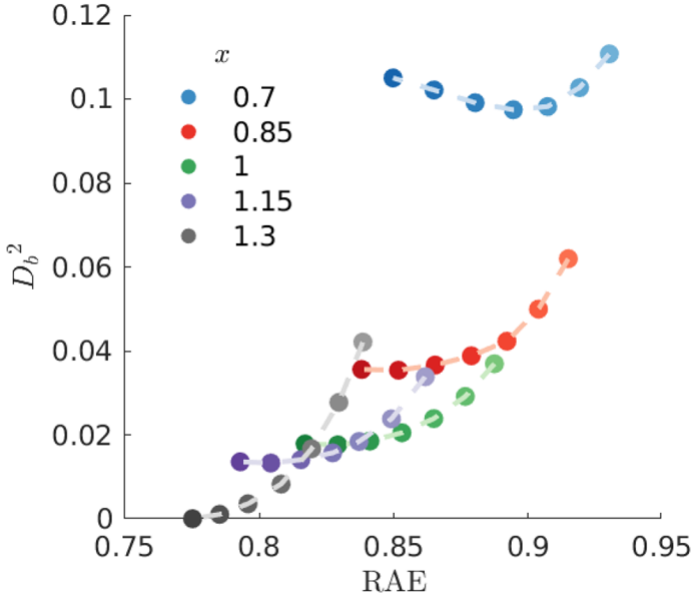
Square distances *D*_*b*_^2^ (eq 24) of the weights from a series of different optimizations relative the best weights with , plotted against the relative absolute error (RAE). Each shade represents a different *t*_0_ ranging from −38 to −14 fs (the darker the shade, the earlier the *t*_0_ shift).

In Figure 7, one can see there is little variation from the *best* set of weights if one examines solutions with more negative *t*_0_ shifts or larger *x*. The cluster of very good optimizations are all found within a small radius of *D*_*b*_^2^ from the optimal solution, and have RAE’s in the range 0.77–0.82 with only a slight reweighting of similar trajectories. The solutions are further removed when *x* = [0.7,0.85], which is a consequence of the optimization redistributing weight between ring-closed trajectories and selecting a different set of ring-opening trajectories. In addition, we note that the better optimizations (as measured by their RAE) tend to exhibit a larger variance in their set of weights, this can be seen in SI Figure S10.

### 5.2 CS_2_ Photodissociation (UED)

**Figure 8:**
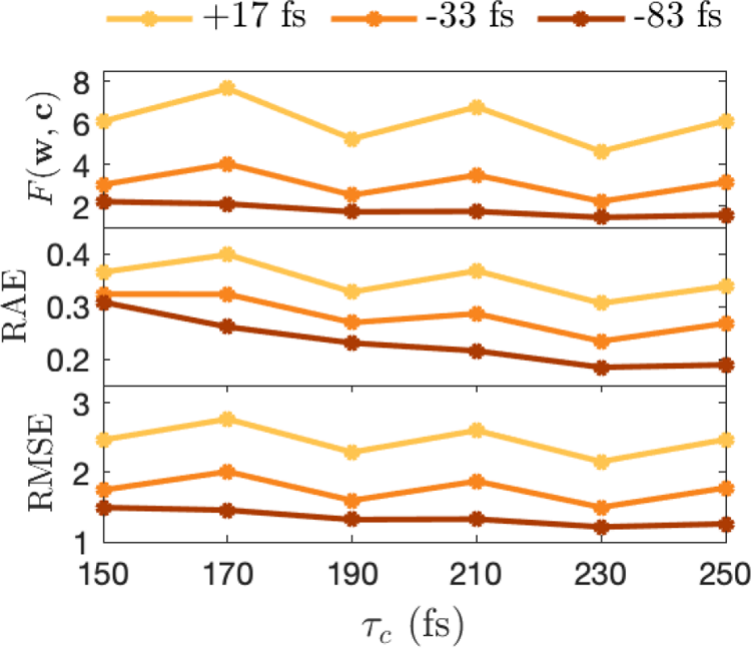
Convergence of the step one optimization with respect to *t*_0_ and τ_*c*_. The panels show the convergence of the target function *F*(**w**, **c**) (top), the RAE (middle), and RMSE (bottom).

**Figure 9:**
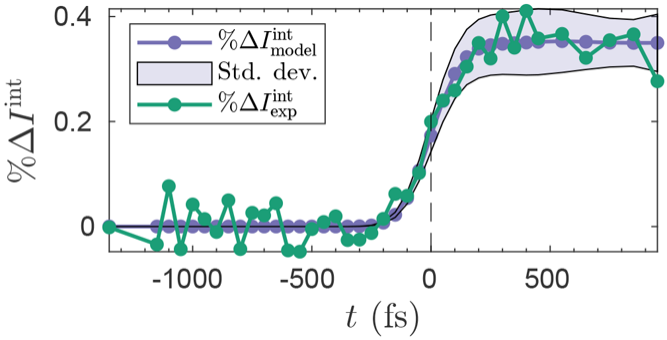
Final result for the step one optimization against the integrated intensity %Δ*I*_model_^int^ (eq 20 with equal weights). The model is convoluted with a Gaussian with τ_*c*_ = 230 fs (fwhm) and the optimized value of excitation fraction is γ = 3%. The shaded area indicates the standard deviation of the ensemble of trajectories from the model calculated assuming equal weights.

We now turn to the more difficult case of CS_2_, where the resolution forces us to employ the two-step optimization procedure. The first step of optimization yields the optimal global parameters . Figure 8 shows that not only does *t*_0_ = −83 fs and τ_*c*_ = 230 fs result in the lowest value of *F*(**w**,**c**), but also that the two complementary error measures RAE and RMSE confirm this. These parameters also yield smoother convergence across all values of τ_*c*_ in comparison to other values of *t*_0_=[+17,–33]. The resulting final best fit in step one is shown in Figure 9. The model tracks the experimental data closely, especially in the important region around *t* = 0, while at other times the rather scattered experimental data is contained with one standard deviation of the model, with the standard deviation calculated on the whole ensemble of trajectories relative the equally weighted “average” model. Also note the significant noise floor in the experimental data evident for *t* ≪ 0. This emphasizes the importance of a robust method for fitting, inverting, and interpreting the experimental data.

For the second step of the optimization, we take the global parameters determined in step one as the starting point for the unconstrained determination of the TBF weights **w**. We also repeat the procedure for three different values of *t*_0_ to check that the optimizations are consistent. During optimization, the predetermined value of the global parameter γ is allowed to readjust. The value should change little but if it does change significantly this may indicate that the values from step one are suboptimal.

**Figure 10:**
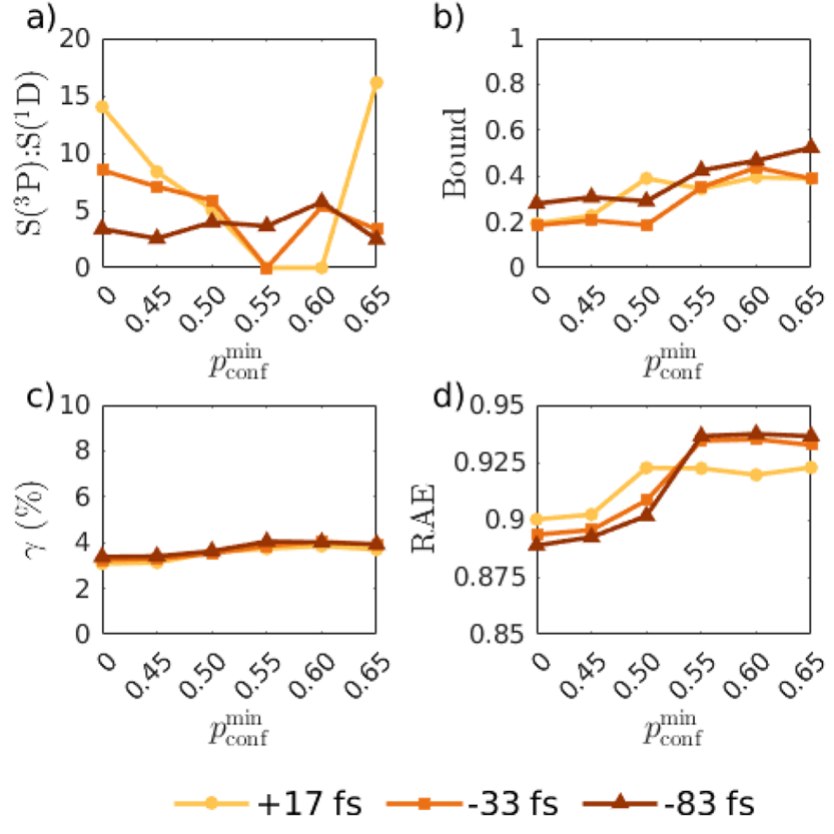
Convergence in CS_2_ optimization (step two) as a function of confidence threshold *p*_conf_^min^ for three values of *t*_0_, showing a) branching ratio, b) bound fraction at 1 ps, c) excitation fraction γ, and d) relative absolute error (RAE).

The weighting of the diffraction signal with the confidence matrix, , is crucial to achieve a sensible fit, with significantly poorer results obtained without it. To investigate this effect, the optimization was repeated for several values of the confidence matrix cutoff threshold, *p*_conf_^min^∈[0, 0.45, 0.50, 0.55, 0.60, 0.65]. As shown in SI Figure S13, increasing the threshold corresponds to hard filtering of the data and lower thresholds correspond to including more data. For example, *p*_conf_^min^ = 0 includes all data, while *p*_conf_^min^ = 0.65 only includes the main feature in Figure 3. Examining the RAE in Figure 10d, it is evident that at all values of *t*_0_, high *p*_conf_^min^ is detrimental to the optimization and *p*_conf_^min^ = 0 yields the best results. This suggests that including all data is beneficial as long as the data is given appropriate confidence weights. This is further confirmed by examining the physical observables resulting from the inversion, as discussed next.

Figure 10a–c shows the convergence of three physical observables as a function of the convergence threshold *p*_conf_^min^ for *t*_0_ = [+17, −33, −83] fs. The observables are not explicitly part of the fit, but are indirectly a function of the optimized weights. The branching ratio between singlet and triplet dissociation product varies significantly over the range of times and confidence thresholds, and convergence to the spectroscopy-derived literature value (≈3) is only obtained for *t*_0_ = 83 fs and then only at low values of confidence threshold *p*_conf_^min^ → 0. In contrast, the convergence of the fraction of bound trajectories at 1 ps is more uniform across the different *t*_0_ values. Values in the range of 20–30%, congruent with spectroscopic results, are observed for the lower confidence thresholds. A similar trend can be observed for the excitation fraction γ, which is comparatively stable across all values of *t*_0_ and *p*_conf_^min^. This indicates that a suitable choice of τ_*c*_ was made and is also reflective of the fact that the value of γ is most governed by the strongest feature in the experimental data (which is also recorded with the lowest noise).

Overall, the best optimization yields the final values , with the convergence of *F*(**w**,**c**) shown in Figure S8 in the SI for reference. The initial guess of γ = 3.0% from step one is reoptimized to 3.4% in the second step, with the comparatively small change suggestive of a stable solution. Examination of the optimized weights show that eight dominant trajectories account for 99.9% of the total weight. Two of these are bound trajectories that make up a total of 28%, three correspond to singlet dissociation, and two to triplet dissociation, which gives a branching ratio of singlet to triplet of 1:3.38.

**Figure 11:**
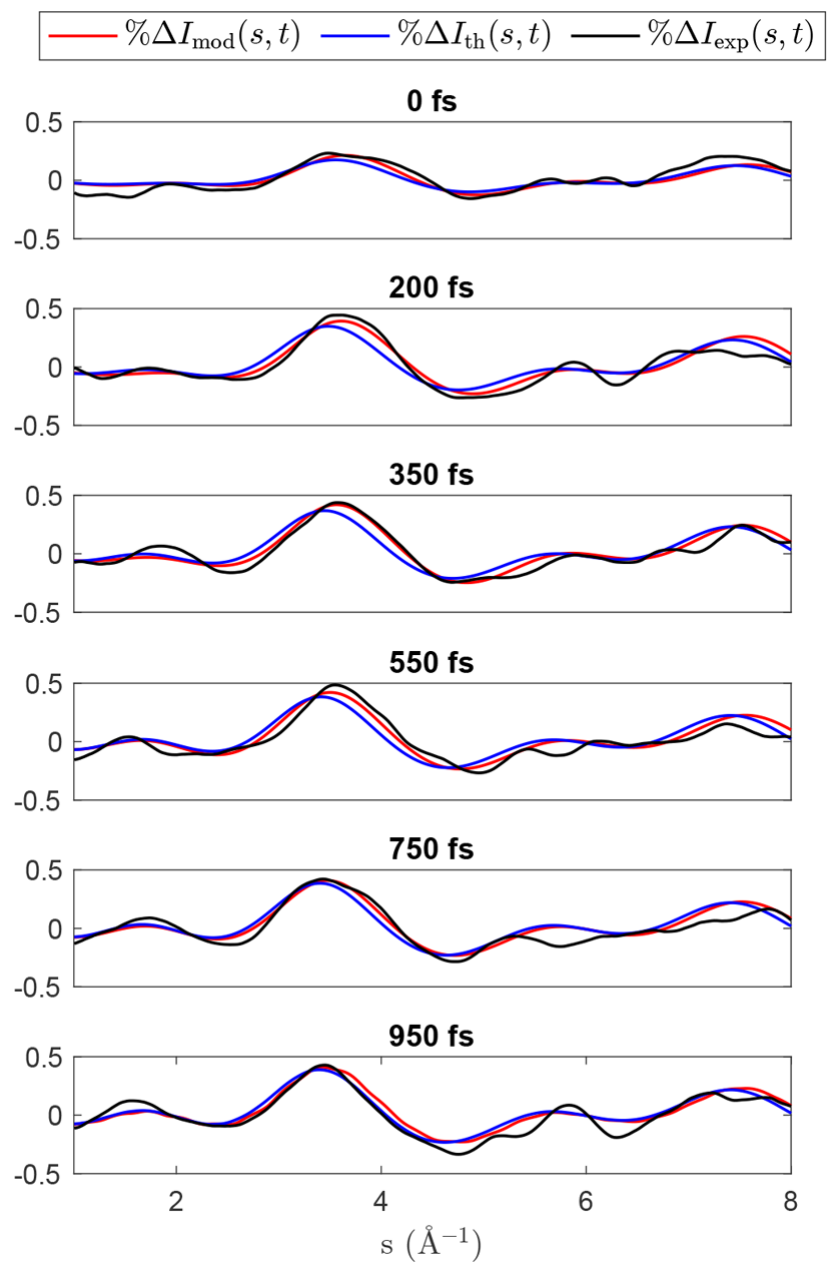
Comparison of the experimental %Δ*I*_exp_(*s*, *t*), unoptimized %Δ*I*_th_(*s*, *t*), and optimized %Δ*I*_mod_(*s*, *t*) signals. The corresponding heatmaps over all time delays can be seen in SI Figure S4.

A summary of the eight trajectories is given in Table 2. The contributions from each of dissociative and bound classes of trajectories to the model signal %Δ*I*_mod_(*s*,*t*) can be seen in SI Figure S7. From this we attribute the onset of the peak below 2 Å^–1^ in the experimental signal %Δ*I*_exp_(*s*,*t*) to dissociation. In Figure 11 we see the result of the optimization on the signal. We observe the optimized model better agrees with experiment in terms of the center and width of the main enhancement correlated with dissociation. In the optimized model, this feature is initially centered at 3.65 Å^–1^ and shifts to 3.43 Å^–1^ at 1 ps, in comparison to 3.55 Å^–1^ and 3.39 Å^–1^ in the unoptimized. Thus, the optimized model is in better agreement with the experimental values of 3.79 Å^–1^ and 3.45 Å^–1^. However, we note that while the model captures the branching ratio and associated time scales correctly, it does not capture the slow leakage of any triplet product before 300 fs. The populations of this minimal basis representation of the dynamics can be seen in SI Figure S5. This is likely a result of the lack of structure and poor confidence in the delayed enhancement below 2 Å^–1^ at earlier times. In Figure 3 it is clear this feature becomes more apparent around 400–500 fs. The associated rise in this features intensity shows good agreement with the rise in the dissociative component of the model (see SI Figure S6); however, the triplet contribution to this intensity takes longer to become apparent. Another issue is that the variation in molecular geometry between singlet and triplet channels is small at early times. The current work only captures the nuclear dynamics, and thus these become difficult to separate. With better resolution of inelastic transitions or by including information from other experiments, the electronic dynamics may become detectable.

**Table 2:** Weights of the Dominant Trajectories in the Best Forward Optimization for CS_2_

Weight (%)	Type
44.7	triplet
17.8	bound
10.4	singlet
10.2	bound
7.42	triplet
4.42	singlet
3.39	triplet
1.67	singlet

**Figure 12:**
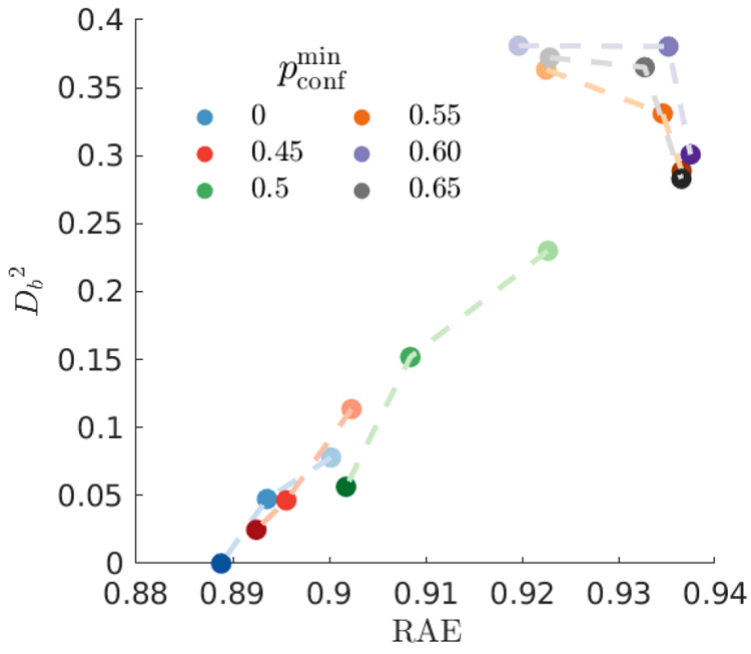
Squared distances *D*_*b*_^2^ (eq 24) of the final TBF weights for CS_2_, examining a series of different optimizations relative the best solution with . The results are plotted with the relative absolute error (RAE) on the *x*-axis. For each value of *p*_conf_^min^, the varying opacity represents a different *t*_0_ shift, ranging from −83, −33 to +17 fs from dark to light, respectively.

In Figure 12 we see that the set of very good solutions, as judged by their RAE value, cluster around the *best* solution as measured by the distance *D*_*b*_ (eq 24). Broadly, this indicates that the optimization procedure succeeds in locating robust global optima. The clustering trend is especially evident when *p*_conf_^min^ ≤ 0.5 and *t*_0_=[−83,–33]. As the value of *p*_conf_^min^ increases and *t*_0_ is shifted toward earlier times, the distances increase. These deviations at high values of *p*_conf_^min^ are explained by the fact that the bound population at 1 ps is overestimated due to the omission of a distinct feature just below 2 Å^–1^ with a delay in the onset of the characteristic of dissociation (see Figure 3). As with CHD, we see that the better optimizations (as measured by their RAE) also exhibit a larger variance in their set of weights, this can be seen in SI Figure S9.

It is worth noting that for the best *t*_0_ and for each confidence threshold, the optimization converges to some combination of the same subset of 16 trajectories. While only 8 of these are significant in the best optimization where , generally the weights of these trajectories show little variation as a function of the confidence threshold. Some are reweighted significantly, for example the dominant triplet trajectory with a weight of nearly 45% only appears in the set of trajectories once the confidence threshold is lowered and more data included. The same is true of a singlet trajectory which sees its weight increase 0% → 4.5% as the confidence threshold is decreased. The opposite effect is seen for a triplet trajectory and a bound trajectory. As more data is included, these trajectories see their weight decrease 20% → 7.5% and 32% → 18%, respectively. Thus, the contribution of some trajectories can be overestimated and the contribution of others underestimated when the optimization is based on high confidence thresholds, i.e. smaller data sets.

A closer examination of the distribution of trajectory weights as a function of *t*_0_ shows that the relative distribution between singlet and triplet trajectories is sensitive to *t*_0_. This is not surprising given that singlet and triplet dissociation is separated temporally, with the singlet dissociation appearing earlier. Shifts in the temporal alignment of experiment and model thus forces the optimization to try to compensate by changing the relative composition of the singlet *vs* triplet dissociation.

Finally, using the iterative weight sampling procedure outlined in the SI, we can confirm that convergence to the global minimum is achieved for the parameters  (for further details, see the SI).

## 6 Conclusions

In this paper, we present a forward optimization method for the inversion of time-resolved data and evaluate its performance on data from actual experiments. The method matches experimental data to a model molecular wave function by optimizing the weights of trajectory basis functions (TBFs). The TBFs arguably provide the most physical constraints for molecular systems far from equilibrium of any inversion method in existence, and ensure that continuity relations are fulfilled. The method can be applied to any type of experiment for which observables can be calculated from the model wave function and to any molecular system for which a basis of trajectories can be generated.

Notably, the TBFs ensure a physically sensible solution even when the inversion is severely underdetermined by the available data and when the data has limited temporal, spatial, or energy resolution (for instance a limited *q*-range in scattering experiments). Importantly, we demonstrate that our approach is robust for noisy data and show that using a confidence matrix for the experimental data is far superior to excluding noisy data from the inversion. For very noisy data, we use a two-step optimization which first optimizes global parameters and then the model wave function. Of course, one must ensure that the TBFs are of good quality, since in the absence of overlap between the space probed by the experiment and the space spanned by the TBFs, meaningful inversion is not possible. Going forward, one could possibly explore a “*theory mapping*”, analogous to the apparatus mapping already implemented, to compensate for systematic shortcomings in the TBFs, for instance by introducing temporal scaling in the TBFs.

We apply the method to ultrafast scattering data for the molecules CS_2_ and CHD. Good agreement with the experimental data is found, and the model performs well also on additional quality measures introduced to evaluate the model wave function and the stability of the solution. The appraisal of the solution includes comparison to data *not* included in the inversion, with the optimized model reproducing key physical properties such as the branching ratio of ring-open to ring-closed product molecules for CHD and the ratio of singlet to triplet dissociation products in CS_2_, derived from spectroscopic measurements. This provides an important sanity check on the inversion and minimizes the risk for incidental agreement between the model and the experimental data. We also note how the inversion method has allowed us to identify key dynamic motifs that contribute to the signal. For CHD, the method disentangles three pathways involved in the ring-opening and for CS_2_ it identifies a key marker of dissociation in the signal. Notably, the small number of representative trajectories in the final model, for both molecules, reflects the limited resolution (*q*_max_/*s*_max_) in the experiments.^93^

Ultrafast experiments tend to include theory and simulations as part of their analysis. The current approach fits naturally into this workflow by allowing the discrepancies between theory and experiment to be identified while providing a robust interpretation of the experimental data. The forward optimization analysis can be performed at small additional cost and provides a bridge between the theory and the experiment, and introduces a systematic strategy for addressing potential shortcomings in simulations.

In future work, forward optimization should be applied to other observables, and, importantly, to multimodal data sets (e.g., combining photoelectron spectroscopy and scattering data). This is will allow a broader range of information to be retrieved in the inversion and will also reduce the risk of overfitting and more clearly highlight any shortcomings in the theoretically derived TBFs. For instance, spectra are sensitive to electronic populations while scattering experiments primarily, but not exclusively, detect the molecular geometry.^12,53,61,100,101^

For scattering experiments specifically, higher quality data will potentially allow for the inversion to include for state-specific scattering,^81,85^ inelastic effects,^102,103^ coherent mixed scattering,^104^ and, finally, alignment effects.^105^ Sophisticated analysis of multidimensional, low-noise data is a prerequisite for unambiguous identification of subtle yet important effects in photochemical dynamics, including interferences or passage through conical intersections, and inversion methods are expected to play an increasingly central role in ultrafast imaging. It is clear that inversion methods can provide credible provisional answers and accelerate the overall process of analysis and interpretation.

## Supporting Information Available

Further details on computation and procedure as well as additional comments on convergence (PDF)
