# Supplementary material for: Robust Inversion of Time-Resolved Data via Forward-Optimization in a Trajectory Basis
Source: J Chem Theory Comput. 2023 May 2;19(10):2721–34. doi: 10.1021/acs.jctc.2c01113 (PMC10210245; doi:10.1021/acs.jctc.2c01113)
Supplement: Supplementary file 1 — ct2c01113_si_001.pdf [file ct2c01113_si_001.pdf]

Supplementary Information

Kyle Acheson† and Adam Kirrander*,‡

†*EaStCHEM, School of Chemistry and Centre for Science at Extreme Conditions, University of Edinburgh, David Brewster Road, Edinburgh EH9 3FJ, United Kingdom*

‡*Physical and Theoretical Chemistry Laboratory, Department of Chemistry, University of Oxford, Oxford OX1 3QZ, United Kingdom*

E-mail: adam.kirrander@chem.ox.ac.uk

Phone: +(0)1865 275422. Fax: +(0)1865 275400

1 Limitations of ΔPDF(R,t)

It is common to represent time-dependent electron scattering signals in terms of the modified scattering ΔsM(s,t), defined as,

$$\Delta \text{sM}(s,t) \approx s\frac{I_{\text{on}}(s,t) - I(s)_{\text{off}}}{I_{\text{at}(s)}}. \tag{S1}$$

Here we use the electron scattering convention of denoting the momentum transfer vector as s. $I_{\text{on}}(s,t)$ and $I_{\text{off}}(s)$ refer to the signal at time t and the $t \ll 0$ signal recorded without a pump pulse. In the denominator, we find the background that results from the incoherent summation of the scattered intensity from individual atoms, which is denoted $I_{\text{at}}(s)$. While the signal represented by defined eq S1 allows one to sine-transform the signal into real space, the division by the *theoretically* calculated $I_{\text{at}}(s)$ also results in an absence of error cancellation in comparison to the signal represented as $\%\Delta I_{\text{exp}}(s,t)$. See Figure S1 for lineouts of the ΔsM signal. Thus, we note that for data with poor signal-to-noise ratio, it is often beneficial to perform the inversion procedure in the reciprocal space of $\%\Delta I_{\text{exp}}(s,t)$.

The sine-transform that allows for the direct inversion of ΔsM(s,t) to real space can be defined as,

$$\Delta \text{PDF}(R,t) = \int_{s_{\text{min}}}^{s_{\text{max}}} \Delta \text{sM}(s,t) \sin(sR) \text{e}^{-\alpha s^2} ds. \tag{S2}$$

The resulting pair distribution function, ΔPDF(R,t), reveals the change in the distribution of interatomic distances R with time. Ideally, the limits of integration in eq S2 should extend from $s_{\text{min}} = 0$ to $s_{\text{max}} = \infty$. In reality, for current MeV-UED experiments, one can expect an upper limit $s_{\text{max}} \approx 12$ Å. Moreover, the reliability of the higher range of s values is limited due to the s^{-4},[1] hence a dampening factor of $\text{e}^{-\alpha s^2}$ is included to limit the effect of large s noise on the transform. Care must be taken in the tuning of the value of α, there is a balance between limiting the detrimental effect of noise at high s and ensuring that the range of s included is sufficiently large to accurately reproduce the distribution of interatomic distances. At the same time, the signal is usually not recorded in the very low range of s due to centre-shifting and streaking resulting from

plasma effects in the target. In the event that only a small amount of data is missing from the low range, linear extrapolation to $s = 0$ can be attempted. However, when s_{min} is too large, this can result in inaccurate or erroneous PDFs. In brief, the lack of data at low s amounts to the exclusion of low frequencies in the sine transform, resulting in the PDF being artificially shifted by a near constant term. The experimentally determined PDF will then exhibit interatomic distances below that of the acceptable physical limit, as well as an unexpected increase in intermediate distances at later times. If the size of the transform window is truncated to exclude the region below 1 Å, then the theoretical PDF can be made to match the experimentally determined PDF, as in Figure S2. While one could envision performing our procedure directly in real space for very high quality UED data in the future, due to these reasons, we perform the fitting procedure in reciprocal space on the signal represented as $\%\Delta I_{\text{exp}}(s,t)$. Overall, this is a much more stable alternative.

2 Iterative Weight Generation Procedure

In cases where convergence is not easily achieved through the standard Monte-Carlo approach to weight generation on the interval [0, 1], it may be desirable to sample the total weight configuration space iteratively through a series of configuration space samples increasing in size. This involves defining a series of bounding limits $(L_{\text{bound}}, U_{\text{bound}})$ on the allowed values of the weights. One starts the procedure with a set of bounds that highly constrain the sample space so that the generated initial weights are narrowly distributed around that of the equally weighted case, i.e. where $\mathbf{w} = 1/N_{\text{TBF}}$. The target function is then minimised with respect to each of the N_{init} initial weights in the pool. If required, the number of sets of initial weights N_{init} can be selectively tuned so that convergence of the target function to the minima within the allowed bounds is observed. From this initial sample of the weight space, the sets of initial conditions that give rise to the lowest value of the target function are selected and entered into the subsequent pool of initial conditions that are generated by slightly increasing the bounds $(L_{\text{bound}}, U_{\text{bound}})$ on the allowed values of the weights. This weight sampling procedure is then repeated for a series of bounds, each of which correspond

to relieving some of the constraint on the allowed values and hence amount to increasing the size of the weight configuration space which is sampled. At each step, the best set of initial conditions are selected and inserted into the next iterations pool. One must be careful that the number of initial conditions N_{init} at each iteration is sufficient to allow convergence. Generally, N_{init} should increase as the the bounds are lifted as the size of the weight sampling space is increasing. This is repeated until the bounds are such that the weights are sampled on the interval [0, 1], which corresponds to the whole weight configuration space. By projecting through a series of different sized weight sampling spaces we provide a way of ensuring the sampling of the space is sufficient enough for convergence to correct global minima to be achieved. It also allows for the refinement of the number of sample sets N_{init} so that it is large enough for global convergence without having to blindly select a huge value which may result in a large computational overhead.

3 Data Treatment

3.1 Ultrafast x-ray scattering (UXS)

Data was recorded over the time range $-1 \leq t_e \leq 4$ ps and ranged from $0.925 \leq q \leq 4.175$ Å^{-1}.[2] In the UXS case, we scale the signal according to fluctuations in the beam intensity. With this in mind, the generalised percent difference signal becomes,

$$\%\Delta I_{\text{mod}}(q,t,\gamma) = \frac{100}{1+\beta(t,\gamma)} \left(\gamma(q) \left[\frac{I_{\text{on}}(q,t)}{I_{\text{off}}(q)} - 1 \right] - \beta(t,\gamma) \right), \tag{S3}$$

where $\beta(t,\gamma)$ is given by,

$$\beta(t,\gamma) = \frac{Q^{\gamma}(t)}{Q_{\text{off}}}. \tag{S4}$$

Here Q_{off} and $Q^{\gamma}(t)$ refer to the integrated signal on the detector with the laser off and on respectively. Notice the excitation fraction $\gamma(q)$ now has a dependence on q, this can be seen in Figure S19. The purpose of the explicit dependence of $\gamma(q)$ on q is to account for the non-uniform nature of the scattered intensity across the measured q range, this arises from the long and ex-

tended interaction region. Note, we do not optimise $\gamma(q)$ directly with respect to q. Rather, we optimise a scaling factor x that acts to uniformly scale $\gamma(q)$ across the available q range such that it yields a scaled optimised excitation fraction $\gamma_x(q)$, which is related to the initial estimate of the q dependent excitation fraction $\gamma(q)$ as, $\gamma_x(q) = \gamma(q)x$. The initial guess of $\gamma(q)$ is calculated from Beer-Lambert law. Clearly $Q^{\gamma}(t)$ is excitation fraction and q dependant. The laser-on case is defined as,

$$Q^{\gamma}(t) = 2\pi \int_{q_0}^{q_1} F_{\text{instr}}(q)\gamma(q)\left[I_{\text{on}}(q,t) - I_{\text{off}}(q)\right] q dq, \tag{S5}$$

where $F_{\text{instr}}(q)$ is the instrument response function that accounts for variation in detector sensitivity with q. The limits of integration q_0 and q_1 refer to the radial limits on the detector. The laser off intensity is given as,

$$Q_{\text{off}} = 2\pi \int_{q_0}^{q_1} F_{\text{instr}}(q) I_{\text{off}}(q)\, q dq. \tag{S6}$$

In the event that the intensity is equal for both the on and off pulses, β reduces to zero and eq S3 reduces to the generalised expression in the main manuscript. As outlined in the main text, the percent difference signal is calculated from a series of trajectories assuming the IAM and using the x-ray scattering form factors $f_A^x(q)$.[3] In the case of UXS, the beam is linearly polarised, and so we scale $\%\Delta I_{\text{mod}}(q,t,\gamma)$ by a polarisation factor $\frac{1}{2}\left(1+\cos^2\theta\right)$. Next, the signal $\%\Delta I_{\text{mod}}(q,t,\gamma)$ is convoluted to mimic the temporal resolution observed in experiment, as described in the main manuscript. The duration of the UV pump laser and the x-ray pulse were measured as 60 fs and 30 fs respectively, thus the value of τ_c is fixed to the product of two Gaussian's with the corresponding width as to mimic the instrument response function. Finally, the signal is binned along the temporal axis into bins of size $\Delta t = 25$ fs. Accounting for all of this, the length of the subset of the signal used in the optimisation becomes $t' + 275$ fs.

To limit the effect of data points with lower statistics on the minimisation of $F(\mathbf{w}, \mathbf{c})$, we define the confidence matrix p_{conf} based on the number of photon hits per frame as,

$$p_{\text{conf}}\left(q_i, t'_j\right) = \frac{N\left(t'_j\right)}{N_{\text{max}}} p_q\left(q_j\right), \tag{S7}$$

where $N(t'_j)$ and N_{max} are the number of hits per frame and the maximum number of hits over all frames respectively. The relative accuracy of each point in q is given by $p_q(q_j)$.

3.2 Ultrafast electron diffraction (UED)

Data was recorded over the range of momentum transfer $0.76 \leq s \leq 12.25$ Å^{-1} and over a temporal range of $-1.26 \leq t_e \leq 1.73$ ps.[4] An initial value of $t' = -120$ fs was selected. In the CS_2 UED case, we define the percentage difference signal in a somewhat simpler fashion as,

$$\%\Delta I_{\text{mod}}(s,t,\gamma) = 100\gamma \times \frac{I_{\text{on}}(s,t) - I_{\text{off}}(s)}{I_{\text{off}}(s)}. \tag{S8}$$

We use relativistic electron scattering form factors $f_A^e(s)$ calculated using ELSEPA.[5] As seen in Figure S3, there is little difference between these and the approximate Mott-Bethe form factors calculated from the tabulated x-ray form factors as $f_A^{\text{e}}(s) = (Z_A - f_A^{\text{x}}(q))/s^2$, where Z_A is the atomic number and $f_A^{\text{x}}(q)$ the x-ray factors. Thus, in this case the form factors have little effect on the result of the fit.

Note that due to instabilities in the *ab-initio* electronic structure calculations at very large nuclear separations, some dissociative trajectories crash before they reach $t = 1$ ps. These trajectorties are extended beyond the point of crashing using a harmonic model of the vibrational motion of the CS fragment remaining. The dissociated atom is extended linearly according to its last known velocity. We define dissociation as the point at which a trajectory reaches a C$-$S bond length greater than 3.4 Å, which is the point of no return in the simulations.

Due to a lack of statistics on the electron beam, we define a confidence matrix p_{conf} as heuristically based on estimated experimental standard deviations from a bootstrapping procedure[4], $\sigma(q_i, q'_j)$. The elements of the confidence matrix are normalised as follows,

$$p_{\text{conf}}(q_i, t'_j) = \left(\frac{\sigma(q_i, t'_j)}{\min\{\sigma(q_i, t'_j)\}} \right)^{-1}. \tag{S9}$$

The matrices $p_{\text{conf}}(q_i, t'_j)$ that result from setting $p_{\text{conf}}(q_i, t'_j) \leq p_{\text{conf}}^{\text{min}}$ to zero, where $p_{\text{conf}}^{\text{min}} \in [0, 0.45, 0.50, 0.55, 0.60, 0.65]$ can be visualised in Figure S13. The case where $p_{\text{conf}}^{\text{min}} = 0$ amounts to including all the data points, but weighting them according to the values in $p_{\text{conf}}(q_i, t'_j)$. The higher the value of $p_{\text{conf}}^{\text{min}}$, the more data points are excluded from the optimisation. The largest value of $p_{\text{conf}}^{\text{min}} = 0.65$ amounts to including only a narrow band of the main enhancement seen in the signal.

4 Additional Comments on Convergence

4.1 Initial Conditions

Given the Monte-Carlo nature of the sampling of initial trajectory weights $\mathbf{w}$, the choice in the number of initial conditions N_{init} is crucial. Too small a number of N_{init} risks that the sampling density of the weight configuration space is inadequate for ensuring that the global minimum is found. In Figure S11, we see that the target function converges rapidly as a function of the number of initial conditions N_{init} used for the optimisations. We observe the lowest value of $F(\mathbf{w}, \mathbf{c})$ when $N_{\text{init}} = 250$. However, from $N_{\text{init}} = 15$ onwards, we see that $F(\mathbf{w}, \mathbf{c})$ only changes by $1e{-}10$ between each successive increase in N_{init}. For $N_{\text{init}} = 100$, which is the value of N_{init} we use in unconstrained optimisations, we see that 46 % of the initial conditions within the sample pool converge to a solution within $1e{-}3$ of the best optimisation.

For the best optimisation, the convergence of the value of the target function and the step size at each iteration in the minimisation, can be seen in Figure S12. The value of $F(\mathbf{w}, \mathbf{c})$ decreases rapidly within the first few iterations as the step size increases. By iteration 19, $F(\mathbf{w}, \mathbf{c})$ reaches a value of 12.18, before converging to 12.12 as the size of the step decreases and the global minimum is located.

4.2 Iterative Weight Sampling - CS_2

Due to the flatness of the target function and the presence of many local minima, we determine that the best optimisation $(t_0, \tau_c, \gamma, p_{\text{conf}}^{\text{min}})$ = (83 fs, 230 fs, 3.4%, 0) is the true global minimum through the weight generation procedure outlined in Section 2. We define a set of 35 upper bounding limits U_{bound}, where $U_{\text{bound}} \in [0.075, 1]$. Note that the set of limits is non-linear and the corresponding values can be seen in Figure S14. For each U_{bound} we perform a constrained optimisation that starts by generating a pool of initial values of $\mathbf{w}$. At each successive iteration we take the best three sets of weights from the previous iteration and add them to the pool of new initial conditions. The number of initial conditions N_{init} in the pool is increased after each iteration. The maximum value of N_{init} may require some tuning, here we find a maximum of $N_{\text{init}} = 100$ at $U_{\text{bound}} = 1$ to be sufficient.

Generally, the same set of trajectories are picked out in all the optimisations, although the lower the value of U_{bound}, the more trajectories are given an appreciable weight. However, these trajectories are very narrowly distributed. This is apparent in the lower panel of Figure S15, where one can see how small the variance v_w^2 is for the set of weights that result in the lowest values of $F(\mathbf{w}, \mathbf{c})$. This can be explained as by forcing a broader distribution in the number of trajectories selected through placing significant constraint on the optimisation, the trajectories are all given a weight close to the limit of U_{bound}. As the constraint is lifted, the variance within the weights $\mathbf{w}$ increases. This effect can also be seen in Figure S18, which shows the trajectory weights as a function of U_{bound}. Once the value of $U_{\text{bound}} = 0.45$ is reached, the weights converge and the optimisation finds the minimal basis representation of the dynamics needed to reproduced the experiment, i.e. it distills out the key dynamics from the broad distribution of trajectories. This is also reflected in the upper panel of Figure S15, which shows the distance of the weights for each U_{bound} from the best optimisation where $(t_0, \tau_c, \gamma, p_{\text{conf}}^{\text{min}}) = $ (83 fs, 230 fs, 3.4%, 0).

Figure S16 shows the convergence of key parameters as a function of U_{bound}. The lack of sensitivity to the value of γ is apparent, indicating that the initial guess of γ as determined in the initial t_0 fit is reasonable. The convergence of the bound fraction appears to take a bit longer,

although values remain in the range of 20-30% for the most part. In comparison, the convergence of the branching ratio is significantly slower, the predicted ratio starting off around 1:2 which is in reasonable agreement with literature. However, this quickly gets worse as some constraint on the optimisation are lifted. The effect is due to over-estimation of the contribution of the singlet population, and it is not until there is sufficient freedom that some of this weight is redistribution to several triplet trajectories. The failure of the constrained optimisations to pick out the finer details is not surprising, given the low resolution in the experimental data and that there is some linear dependence between the singlet and triplet contributions to dissociation.

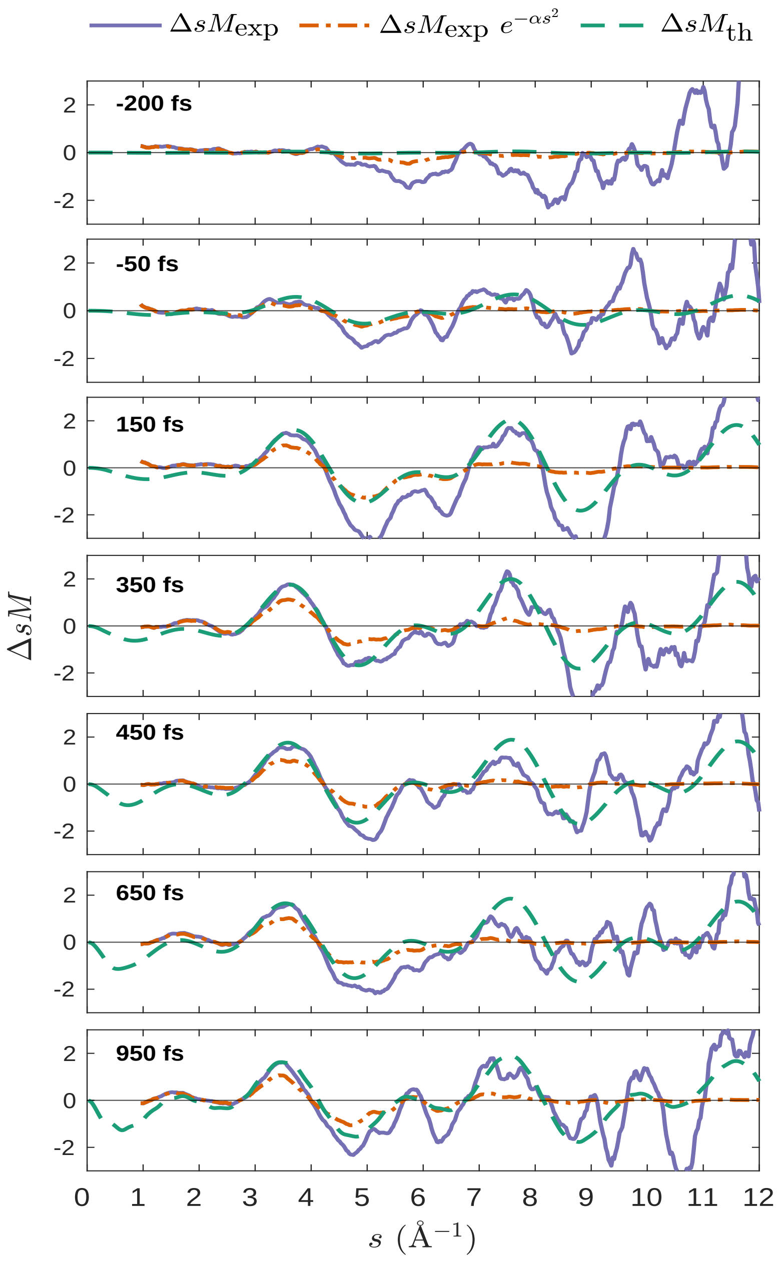

Figure S1 Comparison of experimental and theoretical ΔsM signal as a function of time. Note the inclusion of the dampened experimental $\Delta sMe^{-\alpha s^2}$ which is transformed into real space to obtain the pair distribution function. The noise at high s is heavily reduced at the risk of reducing the intensity of the signal in the intermediate range. Note the absence of experimental data at low values of s.

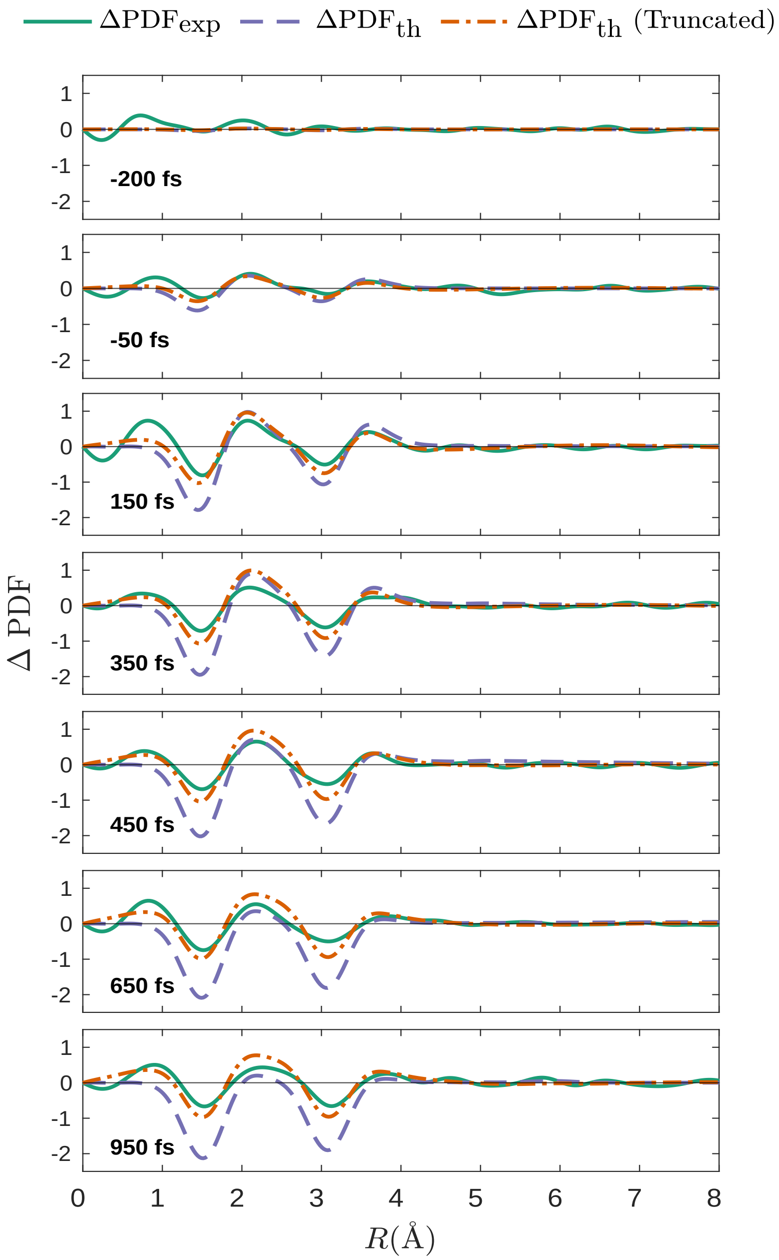

Figure S2 Lineouts of experimental and theoretical ΔPDF. The experimental $\Delta PDF_{\mathrm{exp}}$ and the theoretical ΔPDF_{th} (truncated), which results from truncating the transformation range to exclude the low s region, show good agreement. The absence of the low frequencies from small s yields a vertical shift in the ΔPDFs and an unphysical shoulder at low atomic separations. As a consequence, these two curves indicate the presence of a very short and an intermediate (≈ 2.5 Å) bond length that have no physical basis in CS_2, especially not at large times t. In contrast, the ΔPDF calculated from the theoretical ΔsM which includes the low s data does not suffer these artifacts.

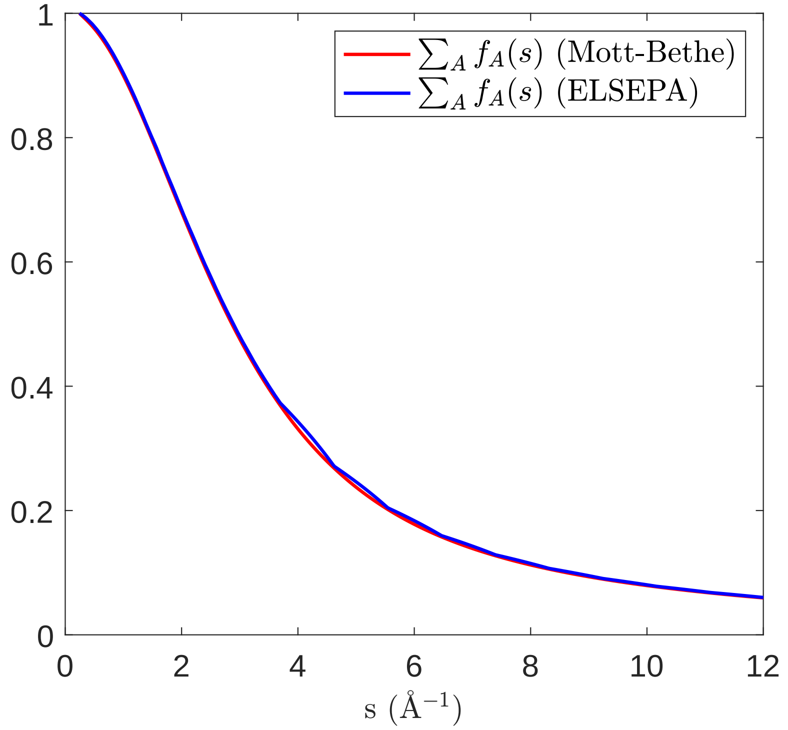

Figure S3 Comparison of the relativistic form factors calculated using ELSEPA.[5] and the approximate Mott-Bethe form factors. Apart from some small undulations in the intermediate s range, little difference can be seen. Note the Mott-Bethe form factors will be unstable as $s \to 0$ due to the s^{-2} term (not seen in the figure).

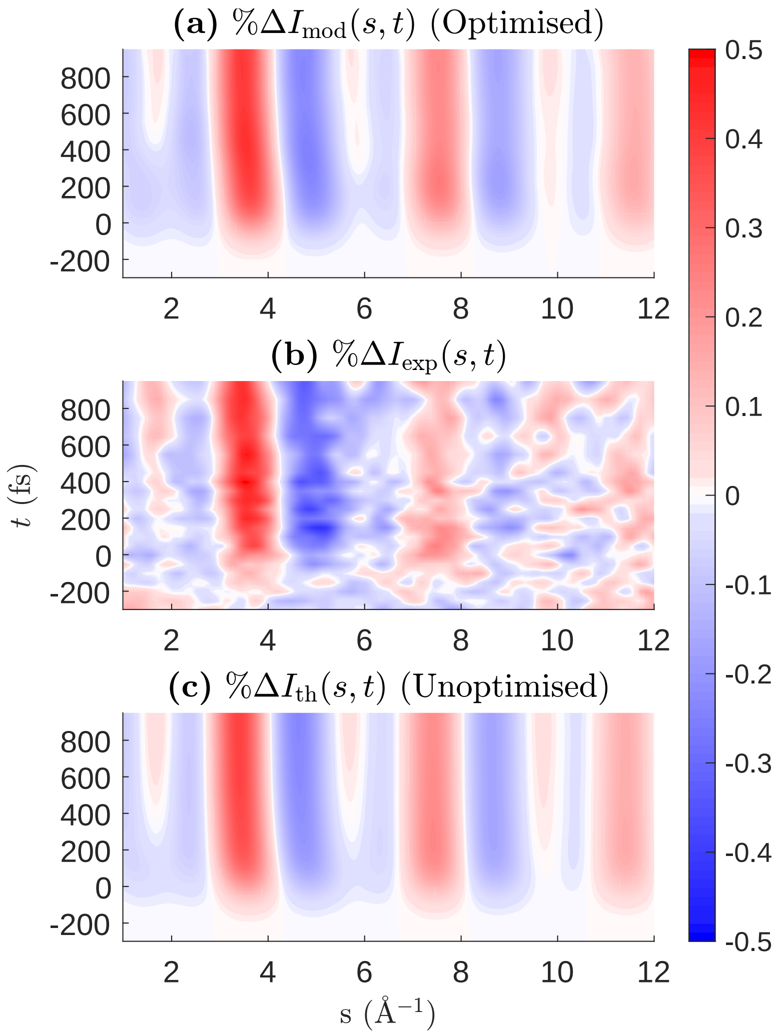

Figure S4 Heat maps of the CS_2 signal before and after optimisation. Optimisation results in a shift of the main enhancement from 3.55 Å^{-1} to 3.65 Å^{-1}, better matching the experimental value of 3.79 Å^{-1}. Both show a delayed enhancement correlated with dissociation just below ≈2 Å^{-1}, first appearing in the unoptimised model just after 200 fs, and in the optimised at 400 fs. The additional delay in the enhancement for the optimised model is likely due to the resolution of the experiment in this region at earlier times, as shown in the main manuscript.

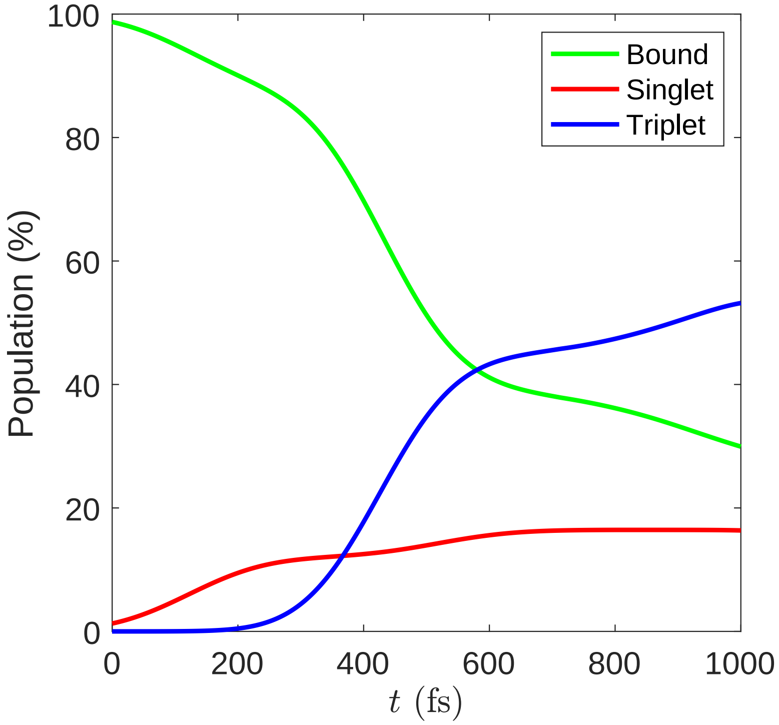

Figure S5 Populations as a function of time for the optimised CS_2 model. Branching ratios and the dominance of the triplet channel at later times agrees with independent spectroscopic experiments. However, the model does not capture the triplet dissociation prior to 300 fs. This is due to the experimental resolution at low s values at these times. In addition, at early times the singlet and triplet channels are very similar in terms of molecular geometry, making them hard to separate in the context of this experiment. Multi-modal approaches including photoelectron data and populations could overcome this.

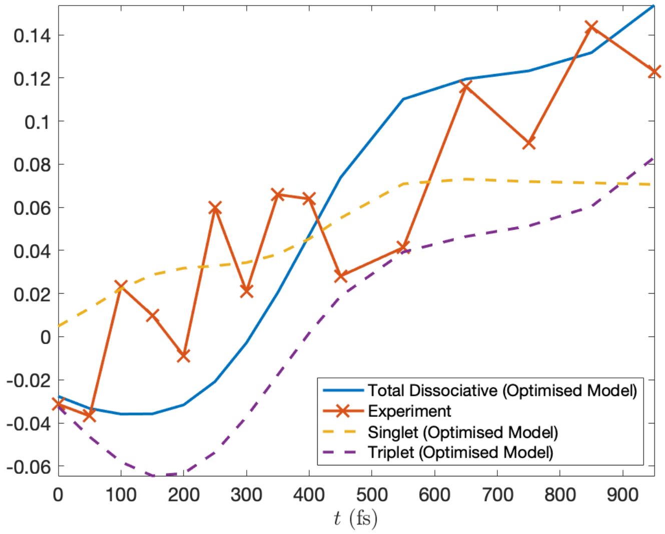

Figure S6 Rise in the delayed enhancement of the CS_2 dataset. Good agreement is seen between the experiment and the combined dissociative channels of the optimised model. However, the triplet contribution of the optimised model takes a while to reach positive values. The shoulder in the experimental signal around 450-550 fs, may be the result of the onset of the bulk of the triplet dissociation. Around this time the singlet contribution to the optimised model levels off and the triplet begins to rapidly increase.

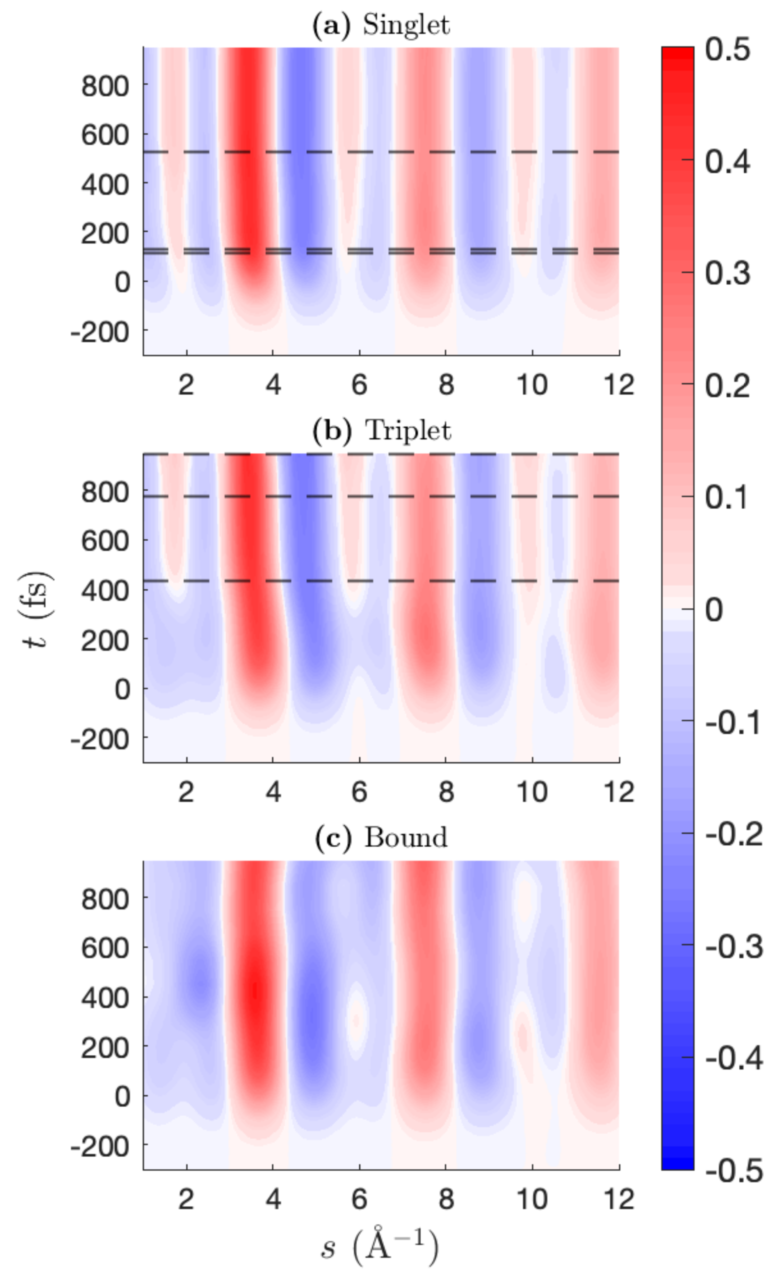

Figure S7 The optimised model CS_2 signal $\%\Delta I_{\mathrm{mod}}(s,t)$ decomposed into its contributions from *a) dissociative singlet*, *b) dissociative triplet*, and *c) bound trajectories*. Dashed black lines represent the dissociation times of the individual trajectories from which the signal is composed. Note the presence of the peak just below 2 Å^{-1} that is delayed in onset and the shift in the main enhancement, these are both correlated with dissociation.

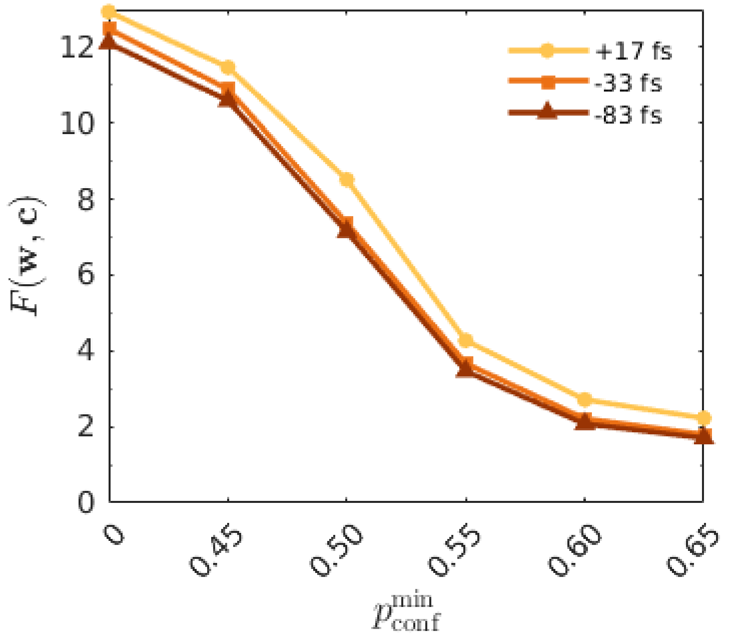

Figure S8 The value of $F(\mathbf{w}, \mathbf{c})$ with respect to confidence threshold $p_{\text{conf}}^{\text{min}}$ at three t_0 shifts. Note that comparisons between different values of $p_{\text{conf}}^{\text{min}}$ hold no meaning as thresholding data points will always lead to a lower value of $F(\mathbf{w}, \mathbf{c})$. The general trend that $t_0 = -83$ fs gives the lowest value is true across all values of $p_{\text{conf}}^{\text{min}}$. This includes $p_{\text{conf}}^{\text{min}} = 0$ which is selected as optimal when the convergence of the statistical measures and physical constants are taken into account, as discussed in the main manuscript.

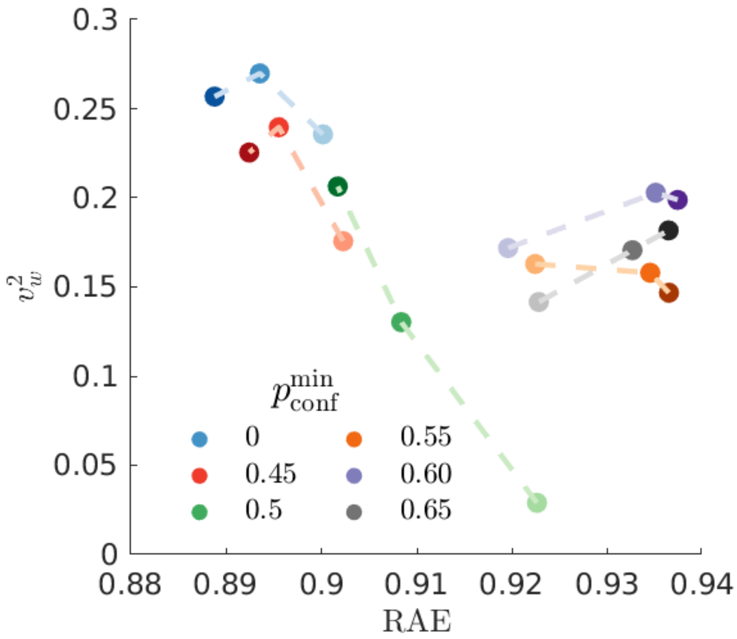

Figure S9 The variance v_w^2 of the best set of weights for a series of CS_2 optimisations for which $p_{\mathrm{conf}^{\mathrm{min}}}$ and t_0 are varied. For each value of $p_{\mathrm{conf}}^{\mathrm{min}}$, the varying opacity represents a different t_0 shift, ranging from -83, -33 to +17 fs from dark to light respectively. The variance of the weights increases for the better optimisations with a lower relative absolute error (RAE).

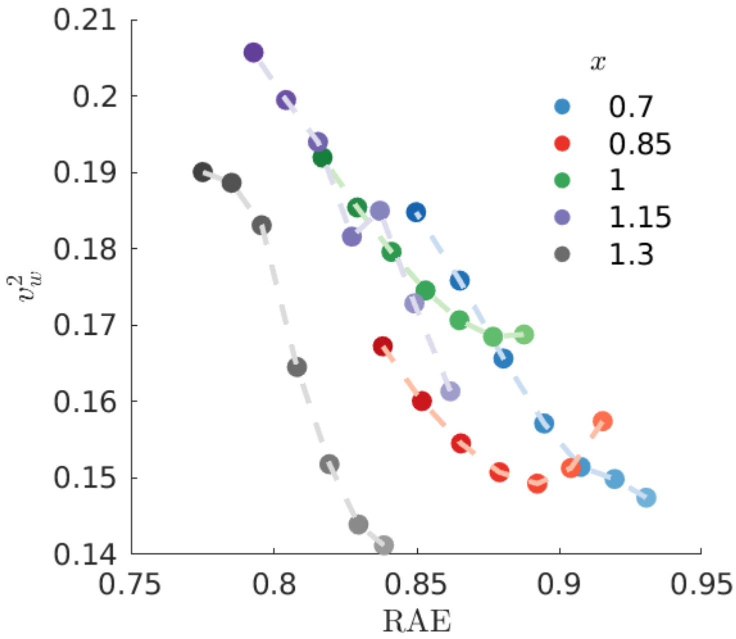

Figure S10 The variance v_w^2 of the best set of weights for a series of CHD optimisations for which the scaling factor x and t_0 are varied. For each value of x, the varying opacity represents a different t_0 ranging from -38 to -14 fs (the darker the shade, the earlier the t_0 shift). The variance of the weights tends to increase for the better optimisations with a lower relative absolute error (RAE).

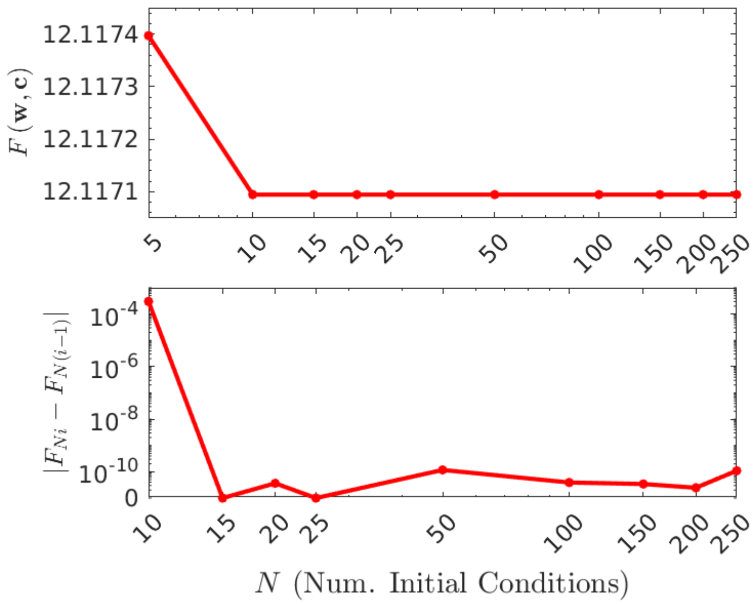

Figure S11 Convergence with respect to the number of initial conditions N_{init} in the Monte-Carlo sampling procedure for the CS_2 reaction. The figure shows the convergence of the minimum value of the target function $F(\mathbf{w}, \mathbf{c})$ *(top panel)* and also the difference between $F(\mathbf{w}, \mathbf{c})$ at each successive sampling density governed by N_{init} *(bottom panel)*. Both are plotted on a logarithmic scale.

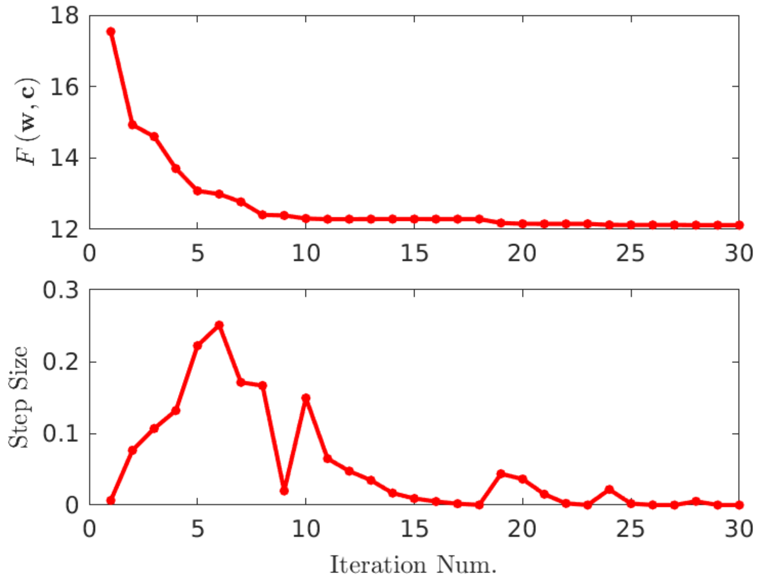

Figure S12 The convergence of the best selected optimisation for CS_2, with respect to the number of iterations. The *top panel* shows the convergence of $F(\mathbf{w}, \mathbf{c})$, and the *bottom panel* shows the convergence of the step size taken along the target function surface at each iteration in the optimisation.

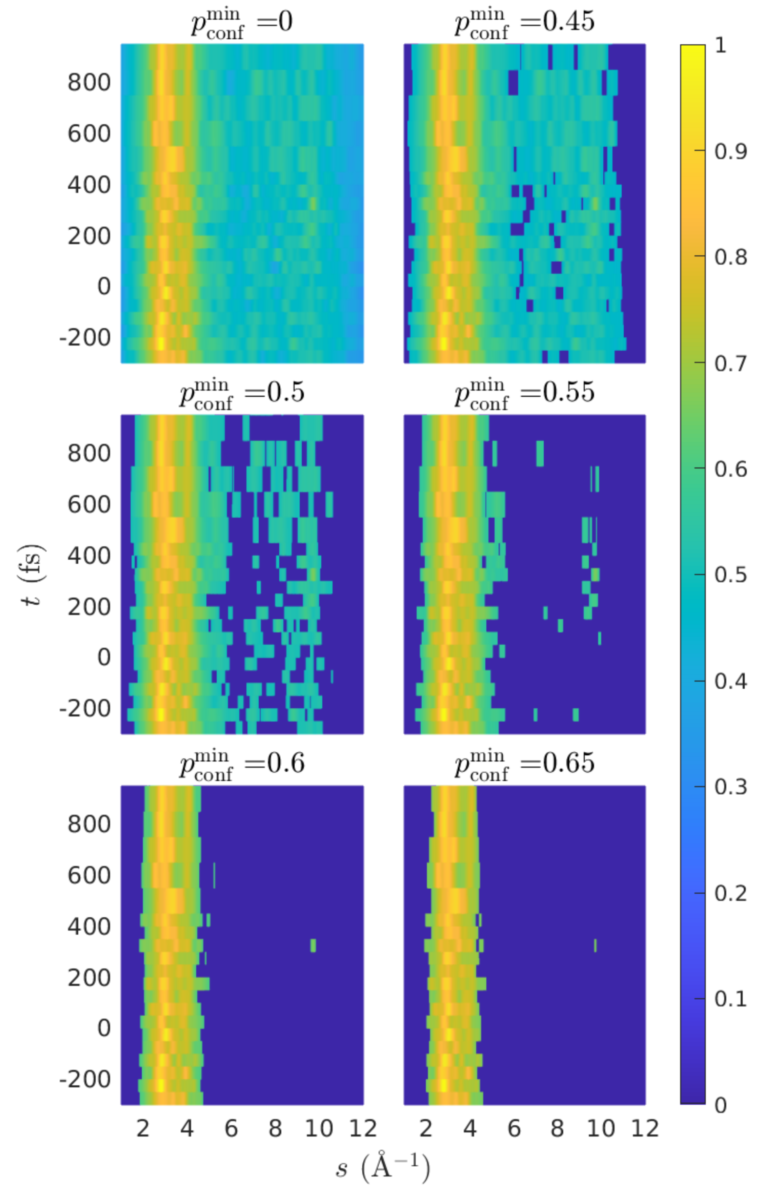

Figure S13 The confidence matrices $p_{\mathrm{conf}}(q_i, t'_j)$ for a series of thresholds $p_{\mathrm{conf}}^{\mathrm{min}}$ used in the optimisation process of CS_2. Each point below the chosen value of $p_{\mathrm{conf}}^{\mathrm{min}}$ corresponds to thresholding out the data by setting to zero.

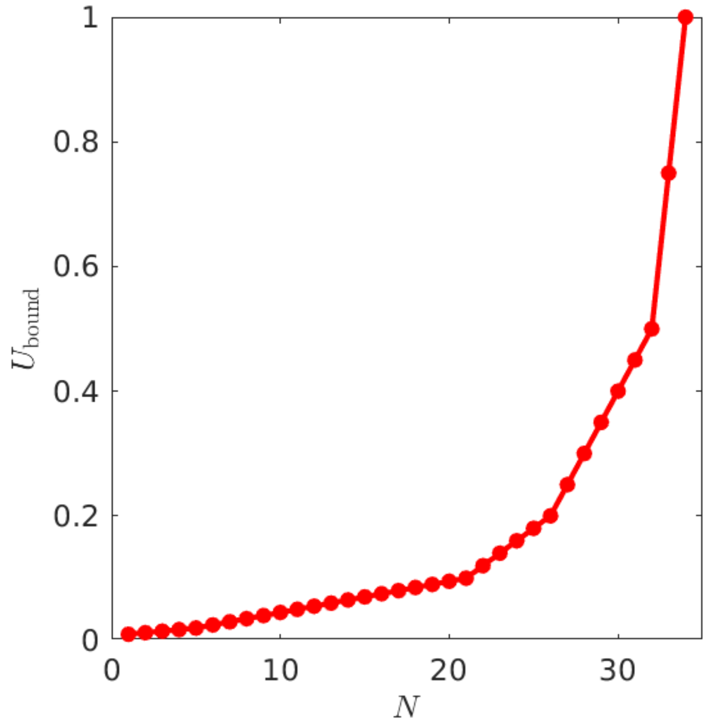

Figure S14 The grid of 35 upper bounds U_{bound} for which a series of constrained optimisations are performed. The end case where $U_{\text{bound}} = 1$ equates to the unconstrained optimisation where $\mathbf{w} \in [0, 1]$.

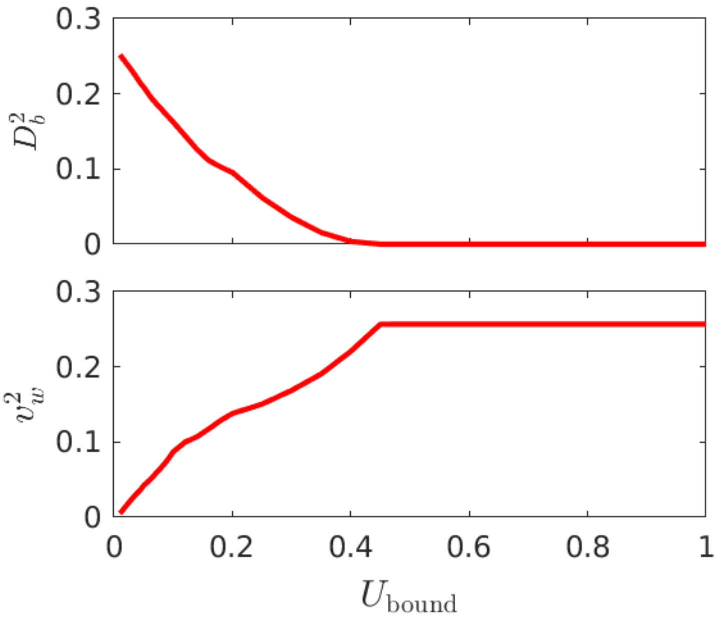

Figure S15 Distance of the weights for a series of U_{bound} values from the best set of weights (top panel) and the variance from the mean weight in each optimisation (bottom panel) i.e. the spread of the weights.

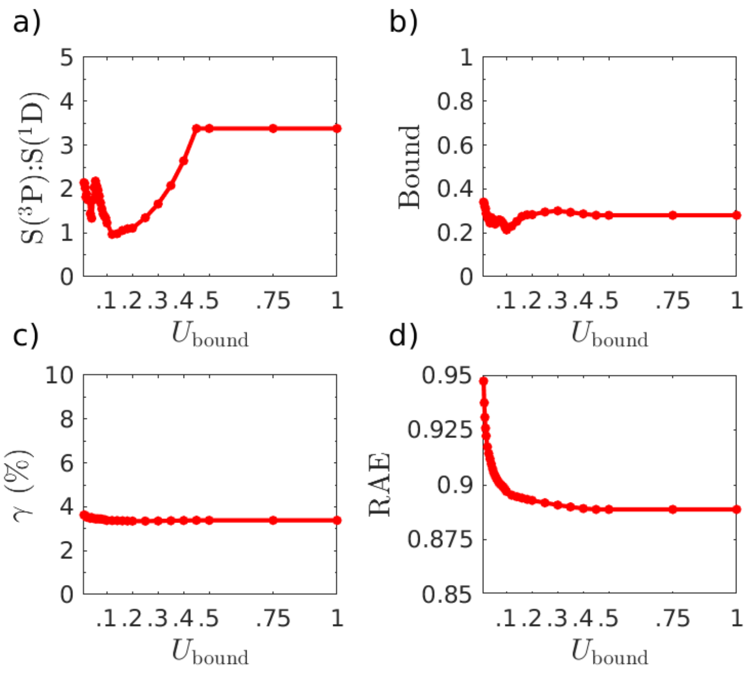

Figure S16 The convergence of a) branching ratio, b) bound fraction at 1 ps, c) excitation fraction (γ), and the relative absolute error as a function of the upper bound on the size of the weight space from which the distribution of initial conditions are generated.

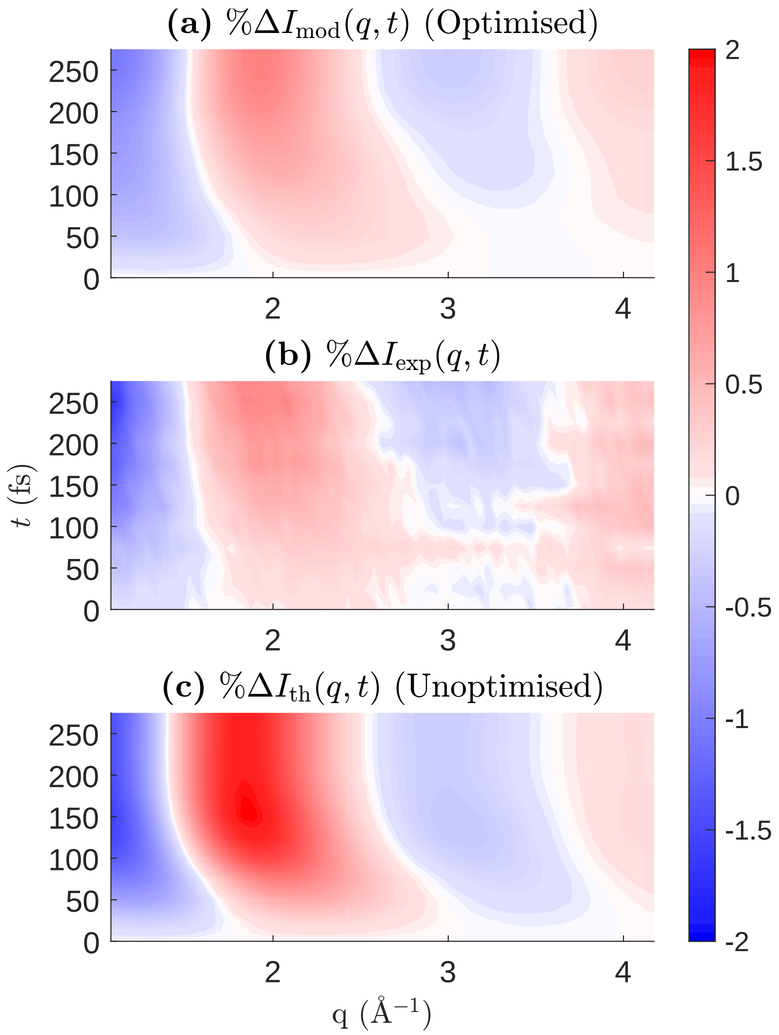

Figure S17 Heat maps of the CHD signal before and after optimisation. The unoptimised signal is more intense, and the main peak between 2-3 Å^{-1} is shifted to slightly lower values of q in comparison to the optimised model and experiment (clearer in the line-outs in the main manuscript).

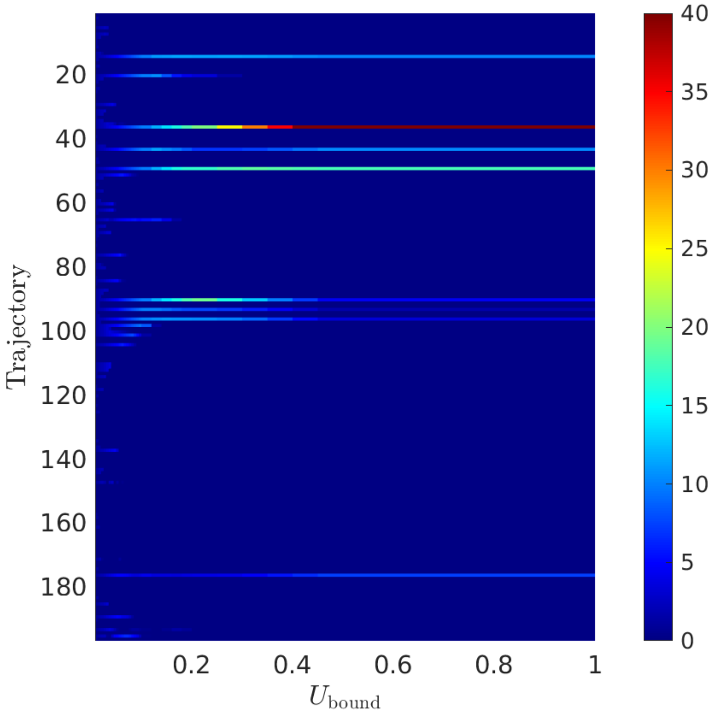

Figure S18 Heatmap of the individial trajectory weights as a function of the upper bound U_{bound} varied in the series of constrained optimisations for CS_2. Highly constrained optimisations result in the weight being distributed more evenly between a broader range of trajectories. As constraint is lifted, the archetypical trajectories are filtered out. $U_{\text{bound}} = 1$ corresponds to the unconstrained case that results in the best fit.

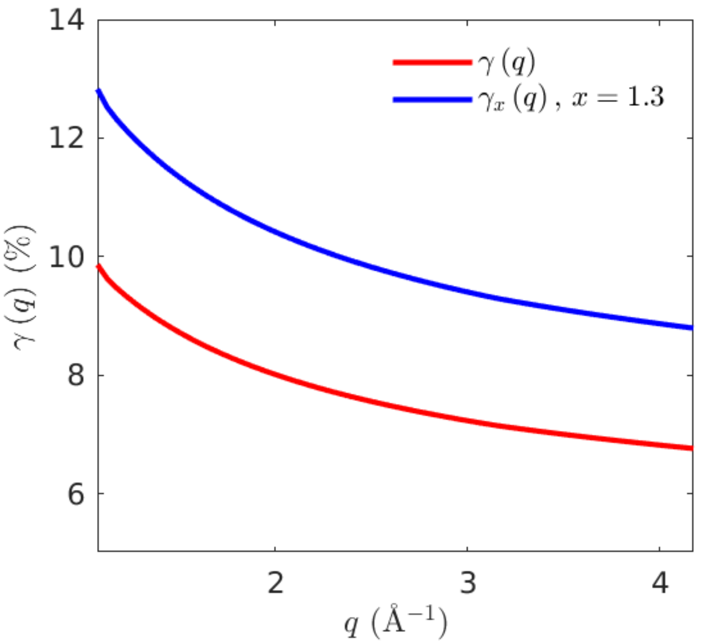

Figure S19 The q dependent excitation fraction $\gamma(q)$ used in the CHD optimisation. This mitigates the effect of different contributions of $\gamma(q)$ across q due to the large interaction region. This is scaled uniformly across q in the target function.

Notes and references

(1) Ma, L.; Yong, H.; Geiser, J. D.; Carrascosa, A. M.; Goff, N.; Weber, P. M. Ultrafast x-ray and electron scattering of free molecules: A comparative evaluation. *Struct. Dyn.* **2020**, *7*, 034102.

(2) Minitti, M.; Budarz, J.; Kirrander, A.; Robinson, J.; Ratner, D.; Lane, T.; Zhu, D.; Glownia, J.; Kozina, M.; Lemke, H.; Sikorski, M.; Feng, Y.; Nelson, S.; Saita, K.; Stankus, B.; Northey, T.; Hastings, J.; Weber, P. Imaging Molecular Motion: Femtosecond X-Ray Scattering of an Electrocyclic Chemical Reaction. *Phys. Rev. Lett.* **2015**, *114*, 255501.

(3) Prince, E., Ed. *International Tables for Crystallography Volume C: Mathematical, physical and chemical tables*, 2006th ed.; Wiley, 2006.

(4) Razmus, W. O.; Acheson, K.; Bucksbaum, P.; Centurion, M.; Champenois, E.; Gabalski, I.; Hoffman, M. C.; Howard, A.; Lin, M.-F.; Liu, Y.; Nunes, P.; Saha, S.; Shen, X.; Ware, M.; Warne, E. M.; Weinacht, T.; Wilkin, K.; Yang, J.; Wolf, T. J. A.; Kirrander, A.; Minns, R. S.; Forbes, R. Multichannel photodissociation dynamics in CS2 studied by ultrafast electron diffraction. *Phys. Chem. Chem. Phys.* **2022**, *24*, 15416–15427.

(5) Salvat, F.; Jablonski, A.; Powell, C. J. elsepa—Dirac partial-wave calculation of elastic scattering of electrons and positrons by atoms, positive ions and molecules. *Comput. Phys. Commun.* **2005**, *165*, 157–190.
